# Synthesis and stereochemical analysis of dynamic planar chiral oxa[7]orthocyclophene

**DOI:** 10.3762/bjoc.22.30

**Published:** 2026-03-11

**Authors:** Yukiho Hashimoto, Yuuya Kawasaki, Kazunobu Igawa, Katsuhiko Tomooka

**Affiliations:** 1 Interdisciplinary Graduate School of Engineering Sciences, Kyushu University, Kasuga, Fukuoka 816-8580, Japan; 2 Institute for Materials Chemistry and Engineering, and IRCCS, Kyushu University, Kasuga, Fukuoka 816-8580, Japan; 3 Faculty of Advanced Science and Technology, Kumamoto University, Kurokami, Kumamoto 865-8555, Japan

**Keywords:** dynamic chirality, medium-sized heterocycle, orthocyclophene, planar chirality, stereochemical analysis

## Abstract

Planar chiral C6-substituted oxa[7]orthocyclophenes were designed and synthesized, and their stereochemical behavior was analyzed. The Kumada–Tamao coupling of the C6-iodo-substituted oxacyclophene enabled the efficient and divergent synthesis of C6-substituted derivatives. The stereochemical analysis of the oxacyclophenes revealed that the iodo- and methyl-substituted derivatives have reasonable stereochemical stability. The planar chirality of the methyl-substituted oxacyclophene was successfully transformed into central chirality by epoxidation without loss of enantiomeric purity.

## Introduction

**Figure 1:**
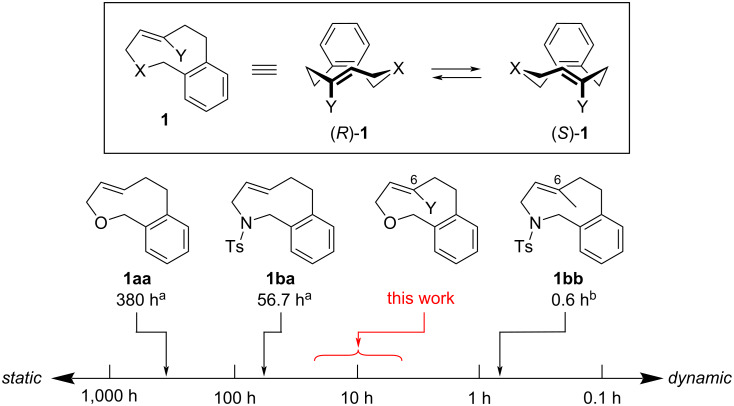
Stereochemical stability of hetera[7]orthocyclophenes. ^a^Half-lives of optical activity at 25 °C in hexane. ^b^Predicted half-lives of optical activity at 25 °C based on the measured value at 5 °C in hexane/EtOH 80:20.

In the course of our study on planar chiral medium-sized cyclic molecules [1–10], we previously synthesized hetera[7]orthocyclophenes **1** having a heteroatom-embedded ansa-chain (X = O or NR) and an (*E*)-alkene moiety (Figure 1) [6,9], and found that the orthocyclophenes exhibit dynamic planar chirality in a wide range of stereochemical stability depending on the differences of the heteroatom and substituents on the (*E*)-alkene moiety [11–12]. The half-lives of the optical activity _opt_*t*_1/2_ of **1aa** (X = O, Y = H) and **1ba** (X = NTs, Y = H) were 380 h and 56.7 h at 25 °C, respectively, indicating that the stereochemical stability of the oxygen-containing cycle is higher than that of the nitrogen cycle: _opt_*t*_1/2_ is one order of magnitude longer. On the other hand, C6-methyl-substituted nitrogen cycle **1bb** (X = NTs, Y = Me) exhibits highly dynamic planar chirality (_opt_*t*_1/2_ = 0.6 h at 25 °C), indicating that a C6-substituent decreases the stereochemical stability: _opt_*t*_1/2_ is two orders of magnitude shorter. This planar chirality is too dynamic to handle the enantioenriched form without loss of enantiomeric purity in standard experimental operations. Based on these trends of the stereochemical stability of the hetera[7]orthocyclophenes, we expected that C6-substituted oxa[7]orthocyclophenes could exhibit reasonable dynamic planar chirality with _opt_*t*_1/2_ approximately 10 h at 25 °C, which is dynamic but can be handled maintaining the enantiomeric purity in careful experimental operations [13–14].

**Figure 2:**
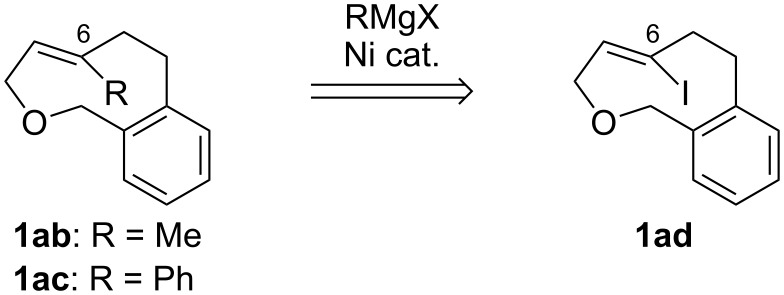
Synthetic plan of **1ab** and **1ac** from **1ad**.

In this study, we have designed, synthesized, and analyzed C6-substituted oxacyclophenes **1ab** (X = O, Y = Me), **1ac** (X = O, Y = Ph), and **1ad** (X = O, Y = I), in which **1ad** was used as the key intermediate for the synthesis of **1ab** and **1ac** via Kumada–Tamao coupling with Grignard reagents (Figure 2).

## Results and Discussion

### Retrosynthesis and synthesis of the C6-iodo-substituted oxacyclophene **1ad**

**Scheme 1:**
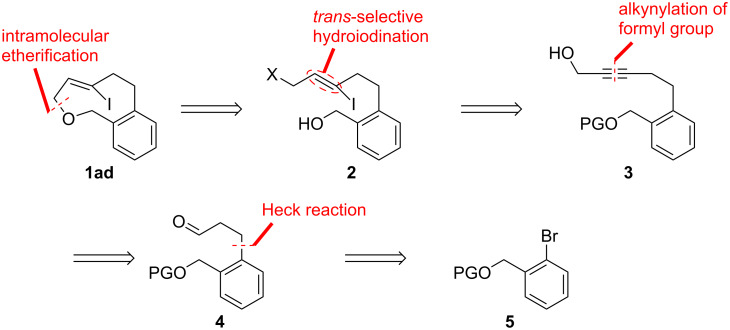
Retrosynthesis of oxacyclophene **1ad**.

We planned to construct the nine-membered cyclic ether skeleton of **1ad** in the final step by an intramolecular Williamson etherification of haloalcohol **2** (Scheme 1). The iodine-substituted *Z*-alkene moiety of **2** was planned to be introduced through a *trans*-selective hydroiodination of propargyl alcohol **3** and the alkyne moiety in **3** can be introduced by an alkynylation of the formyl group of aldehyde **4**. Thus, we started this synthesis from *O*-protected 2-bromobenzyl alcohol derivative **5** for the introduction of the propanal moiety of **4** by a Heck reaction with allyl alcohol.

**Scheme 2:**
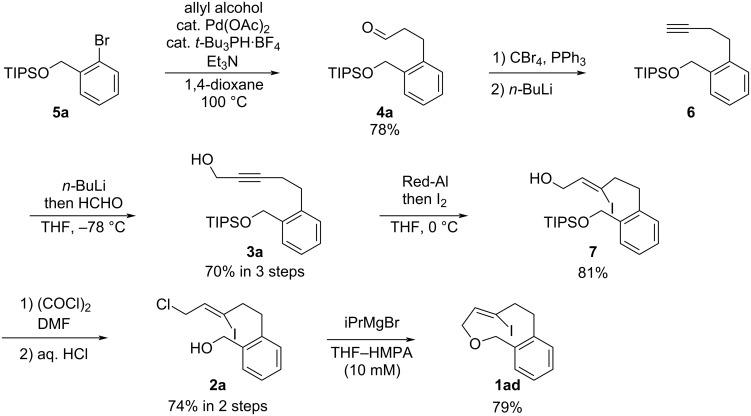
Synthesis of C6-iodo-substituted oxacyclophene **1ad**.

The synthetic route for the C6-iodo-substituted **1ad** is shown in Scheme 2. The Heck reaction of *O*-TIPS-protected **5a** with allyl alcohol in the presence of a palladium catalyst provided the aldehyde **4a** in 78% yield [9]. The reaction of **4a** with CBr_4_ and PPh_3_, followed by treatment with *n-*BuLi, afforded the terminal alkyne **6**. Subsequent preparation of the lithium acetylide using *n-*BuLi, followed by treatment with formaldehyde, afforded propargyl alcohol **3a** in 70% yield over three steps. The reaction of **3a** with Red-Al generated a vinyl aluminum species, which was then treated with iodine to provide the iodinated *Z*-alkene **7** in 81% yield. The hydroxy group of **7** was substituted with chlorine using oxalyl chloride in DMF, and the TIPS group was removed by treatment with hydrochloric acid to afford the chloroalcohol **2a** (74% yield in two steps). After several attempts to cyclize compound **2a** via Williamson etherification, the treatment with iPrMgBr for preparation of the magnesium alkoxide at highly dilute conditions (10 mM) and sequential cyclization successfully proceeded to provide the C6-iodo-substituted oxacyclophene **1ad** in 79% yield.

### Synthesis of C6-methyl and C6-phenyl-substituted oxacyclophenes

**Scheme 3:**
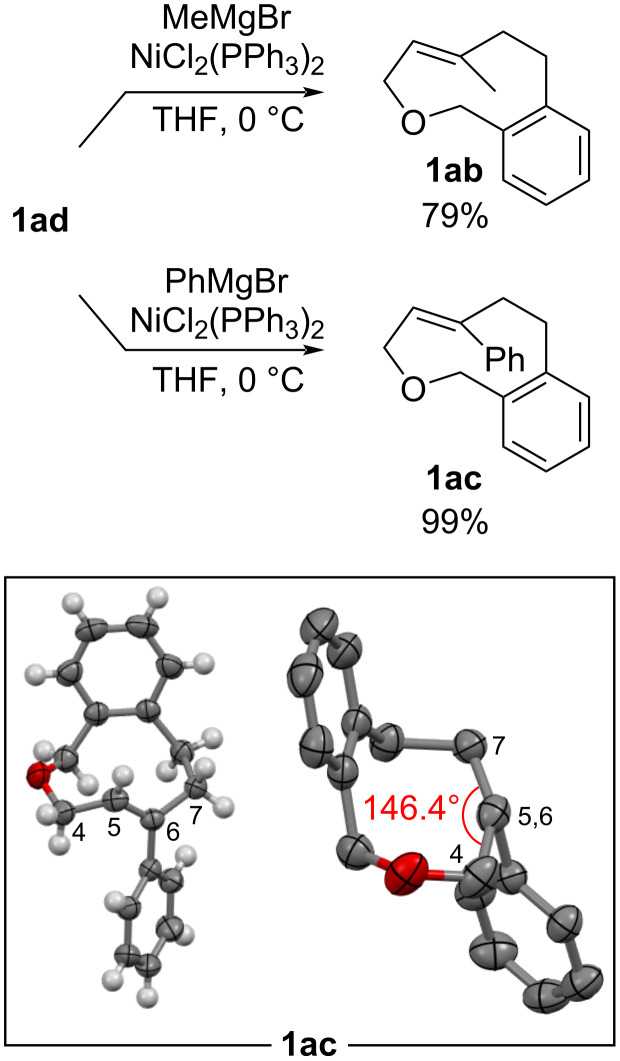
Synthesis of derivatives **1ab** and **1ac**, and ORTEP drawing of **1ac** (ellipsoid set at 50% probability level).

The Kumada–Tamao coupling of compound **1ad** with MeMgBr and PhMgBr in the presence of a catalytic amount of NiCl_2_(PPh_3_)_2_ proceeded smoothly to provide the C6-methyl-substituted **1ab** in 79% yield and the C6-phenyl-substituted **1ac** in 99% yield (Scheme 3) [15–16]. Suitable single crystals were obtained from racemic **1ac**, and its X-ray crystallographic analysis was performed (CCDC 2513894). The solid-state structure of **1ac** shows that the phenyl group on the *E*-alkene is directed antiparallel to the fused benzene ring. The dihedral angle of the alkene moiety (∠C4–C5–C6–C7) of **1ac** is 146.4°, which is distorted by 33.6° from an ideal alkene plane.

### Stereochemical analysis of C6-substituted oxacyclophenes

**Figure 3:**
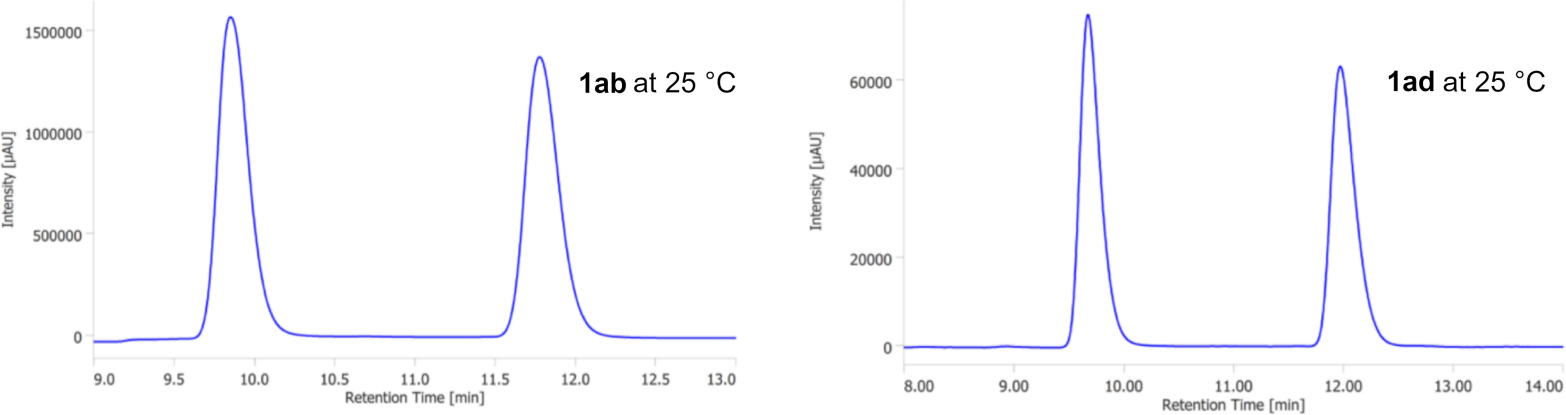
Chromatograms of HPLC analysis of compounds **1ab** and **1ad** using an OJ-H column at 25 °C (OJ-H column, 4.6 mm × 250 mm, eluent: hexane/iPrOH 95:5, flow rate: 0.5 mL/min, monitoring wavelength: 220 nm).

Next, HPLC analyses using a chiral stationary phase (chiral column) of the C6-substituted oxacyclophenes were conducted. The baseline separations of the enantiomers of **1ab** and **1ad** were achieved by using CHIRALCEL OJ-H as the chiral column at 25 °C (Figure 3) [17].

**Figure 4:**
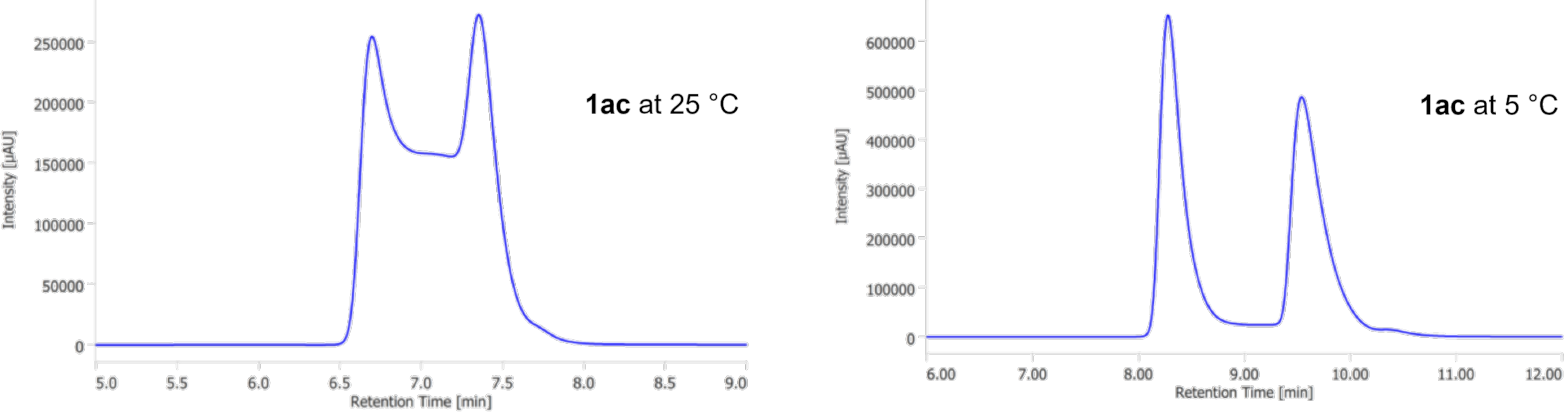
Chromatograms of HPLC analysis of **1ac** (IE column, 4.6 mm × 250 mm, eluent: hexane/iPrOH 95:5, flow rate: 1.0 mL/min, monitoring wavelength: 254 nm).

In sharp contrast to **1ab** and **1ad**, the HPLC analysis of **1ac** showed a plateau between the peaks of the enantiomers at 25 °C, indicating that the interconversion of the enantiomers proceeds on the separation time scale (Figure 4) [18]. The interconversion plateau was still observed in the measurement at 5 °C, albeit only slightly.

**Figure 5:**
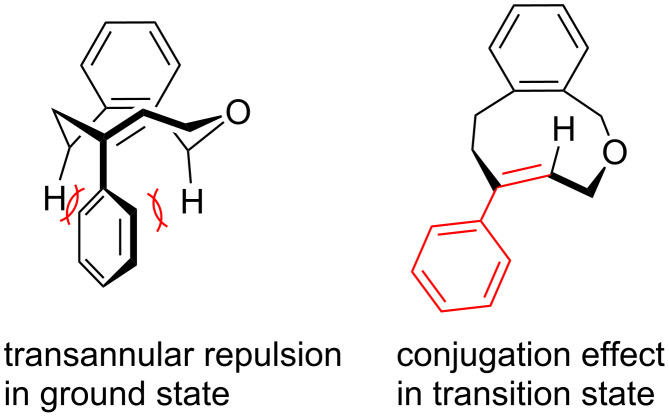
Plausible phenyl-substituent effect on the stereochemical stability of **1ac**.

The phenyl-substituent effect on the stereochemical stability can be understood as a destabilization of the ground state due to the transannular repulsion, and a stabilization of the transition state for racemization by a conjugation effect of the phenyl group with the distorted alkene for a jump-rope rotation-type ring-flip (Figure 5) [10].

**Figure 6:**
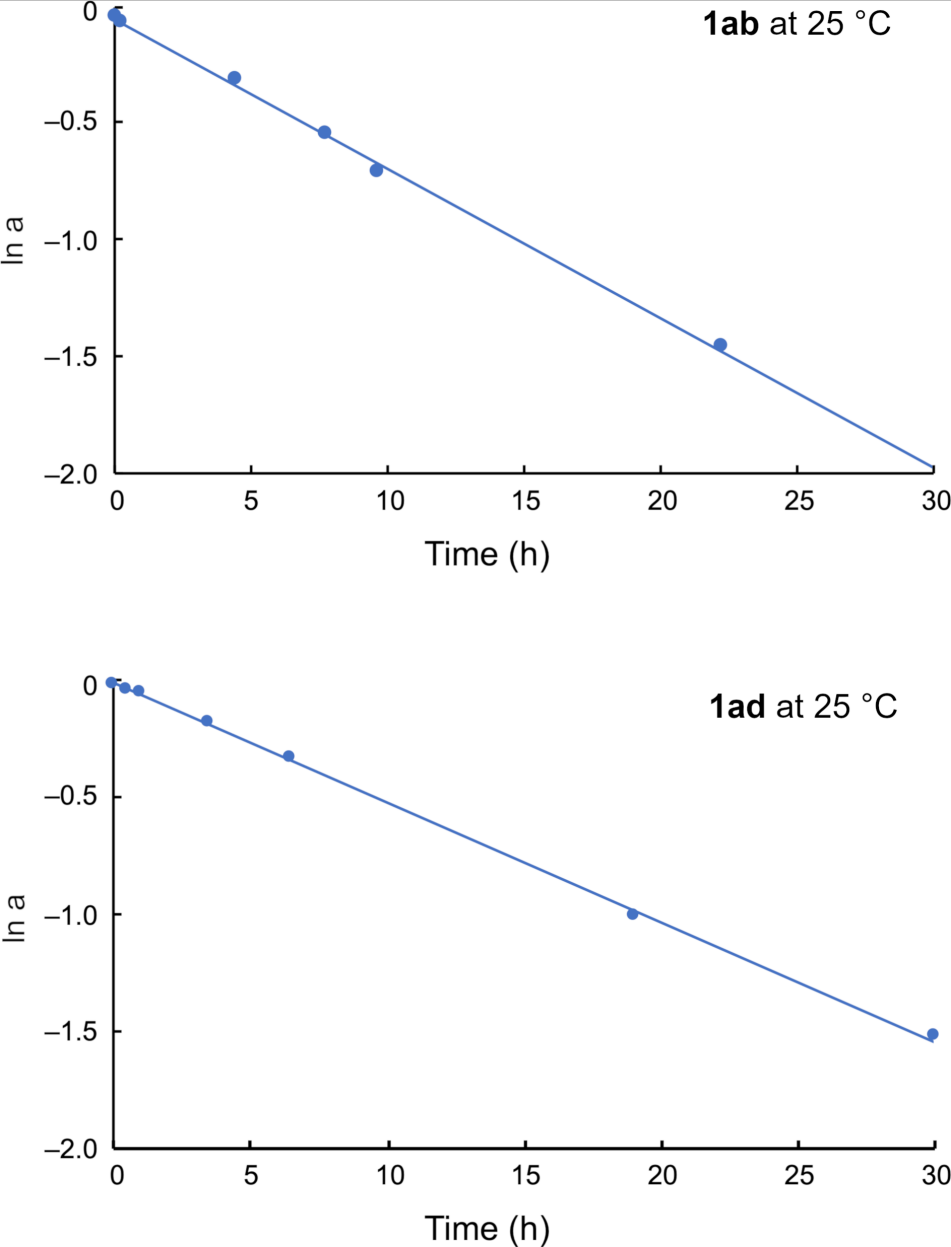
Kinetic measurements for the racemization of **1ab** and **1ad**.

Enantioenriched samples of compounds **1ab** and **1ad** were obtained by HPLC separation using preparative scale chiral columns. We evaluated the stereochemical stability of **1ab** and **1ad** at 25 °C using the eluted samples (solvent: hexane/iPrOH 95:5). The plot of ln *a* (*a* = |*R − S*|/|*R* + *S*|) versus time furnished a straight line, affording the first-order rate constant *k* of racemization. The half-lives of the optical activity of **1ab** and **1ad** are 10.8 h (Δ*G*^‡^ at 25 °C: 24.4 kcal/mol) and 13.6 h (Δ*G*^‡^ at 25 °C: 24.5 kcal/mol), respectively, which are within the predicted range of stereochemical stability (Figure 6).

**Figure 7:**
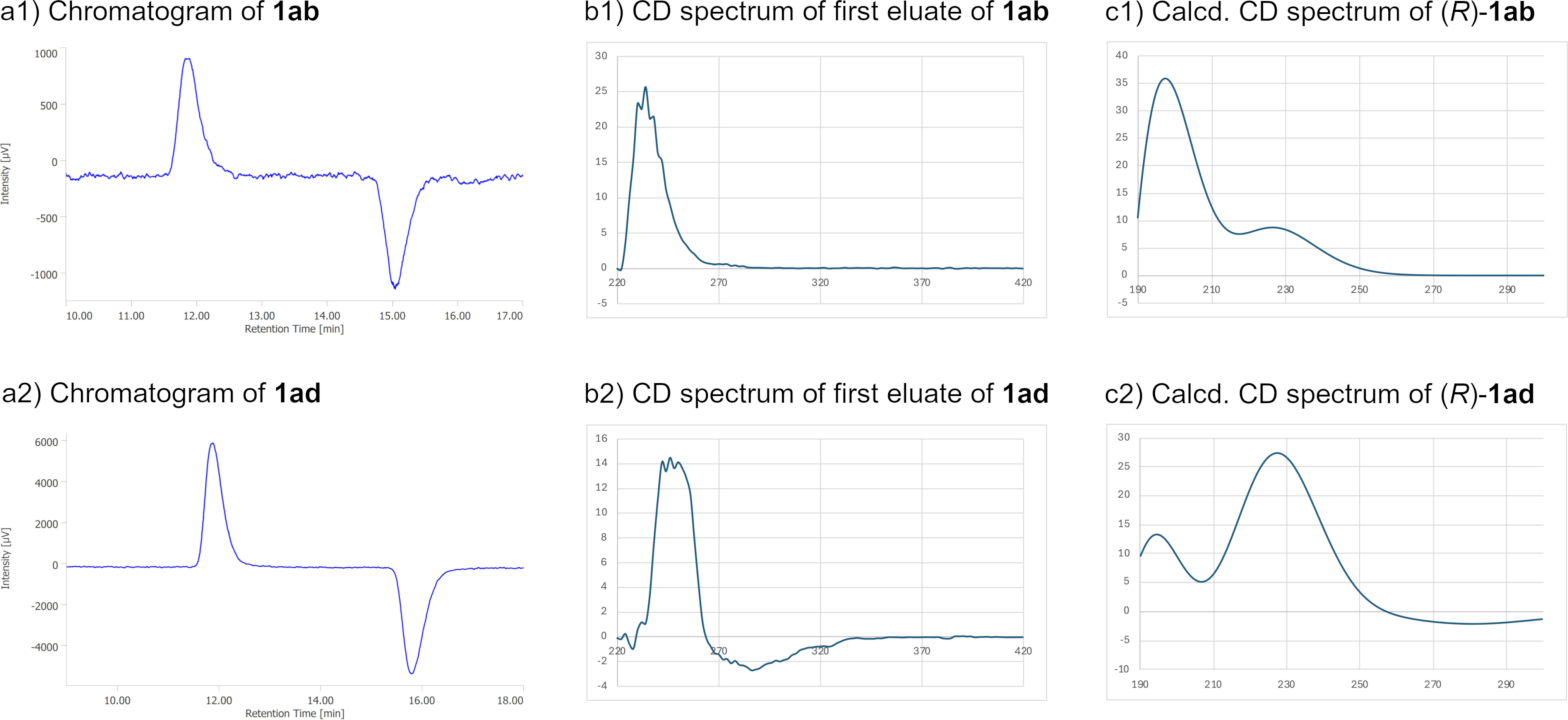
HPLC analysis of **1ab** and **1ad** using a CD detector at 5 °C (OJ-H column, 4.6 mm × 250 mm, eluent: hexane/iPrOH 95:5, flow rate: 0.5 mL/min, monitoring wavelength: 254 nm): a1), a2) chromatograms monitored at 254 nm; b1), b2) CD spectrum of the first eluates in a flow cell; c1), c2) calculated CD spectra using TD-DFT calculations at the B3LYP level using the 6-311G(2d,2p) basis set for C, H, and O atoms and the SDD basis set with the corresponding effective core potential for iodine.

For the determination of absolute stereochemistry of **1ab** and **1ad**, we performed HPLC analysis using circular dichroism (CD) detector [17]. The HPLC analyses with OJ-H column of **1ab** and **1ad** showed similar sense of CD signals, in which the first eluates were detected as a positive signal, and the second eluates detected as a negative signal using the monitoring wavelength at 254 nm (Figure 7). Moreover, the direct CD spectra measurements in a flow cell showed characteristic Cotton effects. The CD spectrum of **1ab** showed a weak positive Cotton effect around 270 nm and a strong Cotton effect around 230 nm, and that of **1ad** showed a weak negative Cotton effect around 290 nm and a strong Cotton effect around 250 nm. Each TD-DFT calculations for (*R*)-**1ab** and (*R*)-**1ad** showed good agreement in shape for the CD spectra of the first eluates, thus we determined that the absolute stereochemistry of the first and second eluates corresponds to the (*R*)- and (*S*)-isomers, respectively.

### Transformation of planar chirality of **1ab** to central chirality

**Scheme 4:**
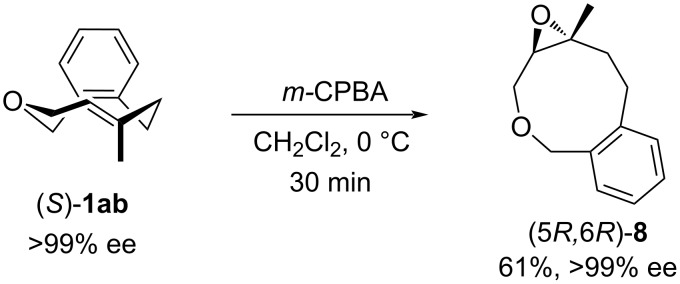
Epoxidation of (*S*)-**1ab**.

The planar chirality of the methyl-substituted oxacyclophene can be transformed into central chirality without loss of enantiomeric purity. For example, (*S*)-**1ab** obtained by chiral HPLC separation using an OJ-H column was treated with *m-*CPBA, in which (*S*)-**1ab** was smoothly consumed at 0 °C within 30 minutus to afford epoxide **8** in 61% yield (Scheme 4). HPLC analysis of the obtained product **8** using chiral column showed an enantiomeric purity of >99% ee. In this reaction, *m-*CPBA can only approach from the outside of the ring skeleton of **1ab**, so (5*R*,6*R*)-**8** should be obtained from (*S*)-**1ab**.

## Conclusion

In summary, we synthesized C6-substituted oxa[7]orthocyclophenes and revealed their dynamic planar chirality. By using the C6-iodo-substituted oxacyclophene as a common intermediate, C6-methyl- and C6-phenyl-substituted oxacyclophenes were synthesized efficiently. The enantiomers of the iodo- and methyl-substituted oxacyclophenes are isolable yet interconvertible at ambient temperature. The phenyl-substituted oxacyclophene showed more pronounced dynamic planar chirality than the iodo- and methyl-substituted derivatives. The planar chirality of the oxacyclophene was successfully transformed into central chirality by epoxidation without loss of enantiomeric purity.

## Supporting Information

File 1 Experimental procedures, characterization data, copies of ^1^H and ^13^C NMR spectra, and optimized geometries of DFT calculations.

File 2 Crystallographic information file of compound **1ac**.

## Data Availability

All data that supports the findings of this study is available in the published article and/or the supporting information of this article.
