# Supplementary material for: Synthesis and stereochemical analysis of dynamic planar chiral oxa[7]orthocyclophene
Source: Beilstein J Org Chem. 2026 Mar 11;22:436–42. doi: 10.3762/bjoc.22.30 (PMC12990439; doi:10.3762/bjoc.22.30)
Supplement: File 1 — Experimental procedures, characterization data, copies of 1H and 13C NMR spectra, and optimized geometries of DFT calculations. [file Beilstein_J_Org_Chem-22-436-s001.pdf]

BEILSTEIN JOURNAL OF ORGANIC CHEMISTRY

Supporting Information

for

Synthesis and stereochemical analysis of dynamic planar chiral oxa[7]orthocyclophene

Yukiho Hashimoto, Yuuya Kawasaki, Kazunobu Igawa and Katsuhiko Tomooka

Beilstein J. Org. Chem. **2026,** *22,* 436–442. doi:10.3762/bjoc.22.30

Experimental procedures, characterization data, copies of ^{1}H and ^{13}C NMR spectra, and optimized geometries of DFT calculations

License and Terms: This is a supporting information file under the terms of the Creative Commons Attribution License (https://creativecommons.org/licenses/by/4.0). Please note that the reuse, redistribution and reproduction in particular requires that the author(s) and source are credited and that individual graphics may be subject to special legal provisions.
The license is subject to the *Beilstein Journal of Organic Chemistry* terms and conditions: (https://www.beilstein-journals.org/bjoc/terms)

Table of contents

1. General information S2

2. Experimental procedures S3–11

3. Copies of ^{1}H and $^{13}C\{^{1}H\}$ NMR spectra S12–20

4. DFT calculations S21

5. Kinetic measurements of the racemization S22

1. General information

All anhydrous reactions were carried out in heat-gun-dried glassware under an argon atmosphere. All reagents were purchased from Kanto Chemical Co. Inc., Fujifilm Wako Pure Chemical Co. Ltd., Tokyo Chemical Industry Co. Ltd., and Sigma-Aldrich Co. LLC. A Mettler Toledo XS205DU instrument was used for measurements. Dry CH_2Cl_2, THF, and DMF were purchased from Kanto Chemical Co., Inc. and used without purification. ^1H NMR and $^{13}C\{^1H\}$ NMR were recorded on a Varian Mercury 300 (^1H NMR: 300 MHz, $^{13}C\{^1H\}$ NMR: 75 MHz) or a JEOL JNM-ECZL-600G (^1H NMR: 600 MHz, $^{13}C\{^1H\}$ NMR: 150 MHz) using $CDCl_3$ as a solvent. Proton chemical shifts are expressed in parts per million (ppm, δ scale) and are referenced to the residual proton in the NMR solvent ($CHCl_3$: 7.26). The carbon chemical shifts are expressed in parts per million (ppm, δ scale) and are referenced to the carbon of the NMR solvent ($CDCl_3$: δ 77.1). The peak multiplicities are given as follows: s, singlet; d, doublet; t, triplet; m, multiplet; br, broad. Optical rotation values were measured on a digital polarimeter (JASCO P2200). Infrared (IR) spectra were recorded on a JASCO FT/IR-4600 instrument with an attenuated total reflection (ATR). High performance liquid chromatography (HPLC) was performed on a system consisting of JASCO CD-2095, JASCO MD-2018, JASCO CO-2067, and JASCO PU-2089 using DAICEL CHIRALCEL OJ-H, CHIRALPAK IE, and CHIRALPAK IH columns. Single crystal X-ray structural analyses were carried out on a Rigaku XtaLAB Synergy-R diffractometer with Rigaku HyPix-6000 area detector using multi-layer mirror monochromated Cu Kα radiation. Melting points (mp) were measured on a Yanaco Micro Melting Point Apparatus. Analytical thin-layer chromatography (TLC) was carried out on silica gel 60 F_{254} (Merck 5715) plates and developed plates were visualized by UV light (254 nm) and by heating on a hot plate after staining with a 4% solution of phosphomolybdic acid in ethanol or a 2.5% solution of *p*-anisaldehyde in ethanol. Silica gel column chromatography was performed using Fuji Silysia FL100D (spherical neutral, particle size 100 μm) or Kanto 60N (spherical neutral, particle size 100–210 μm).

2. Experimental procedures

((2-(But-3-yn-1-yl)benzyl)oxy)triisopropylsilane (6)

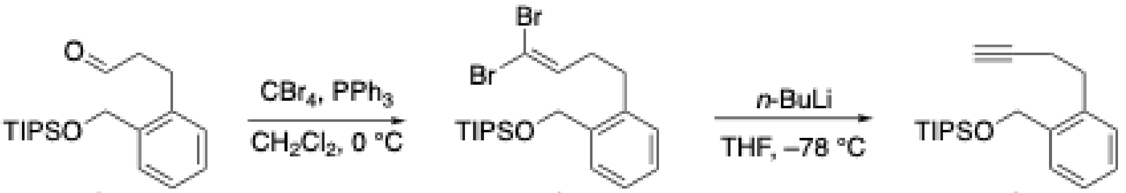

Compound **4a** was prepared by the reported procedure: *Chem. Lett.* **2022**, *51*, 788-790.

To a solution of PPh_3 (11.3 g, 43.2 mmol) and CBr_4 (7.05 g, 21.6 mmol) in CH_2Cl_2 (80 mL) was slowly added **4a** (3.46 g, 10.8 mmol) in CH_2Cl_2 (80 mL) at 0 °C. After the mixture was stirred for 30 min at that temperature, the mixture was concentrated under reduced pressure. To remove insoluble by-products, hexane (50 mL) was added to the mixture and the resulting precipitate was removed by filtration. This process was repeated three times to afford crude product **9** which was used without further purification in the next step. To a solution of crude **9** in THF (100 mL) was added *n*-BuLi (1.30 M in hexane, 17.4 mL, 22.7 mmol) at −78 °C and the mixture was stirred at that temperature for 30 min, then warmed to 0 °C and stirred for additional 30 min. The reaction was quenched with sat. aq. NH_4Cl and extracted with hexane. The combined organic phase was washed with brine and dried over Na_2SO_4, filtered, and the mixture was concentrated under reduced pressure. The residue was purified by silica gel chromatography (hexane/AcOEt 98:2) to afford 3.08 g (83%) of **6** as a colorless syrup.

^{1}H NMR (300 MHz, $CDCl_3$): δ 7.47-7.41 (m, 1H), 7.19-7.26 (m, 3H), 4.85 (s, 2H), 2.87 (t, *J* = 7.7 Hz, 2H), 2.49 (td, *J* = 7.7, 2.6 Hz, 2H), 1.98 (t, *J* = 2.6 Hz, 1H), 1.08-1.22 (m, 21H).

^{13}C{^{1}H} NMR (75 MHz, $CDCl_3$): δ 138.98, 137.45, 128.75, 127.09, 127.06, 126.49, 83.93, 68.80, 63.19, 31.03, 19.80, 18.09, 12.04.

HRMS (FAB, matrix: 3-nitrobenzyl alcohol, positive): Exact mass calcd. for $C_{21}H_{31}O_2Si$ $[M–H]^+$ requires *m/z*: 315.2144, found *m/z*: 315.2147

IR (ATR, cm^{-1}): 3313, 2941, 2865, 1755, 1463, 1381, 1119, 1066, 882.

5-(2-(((Triisopropylsilyl)oxy)methyl)phenyl)pent-2-yn-1-ol (3a)

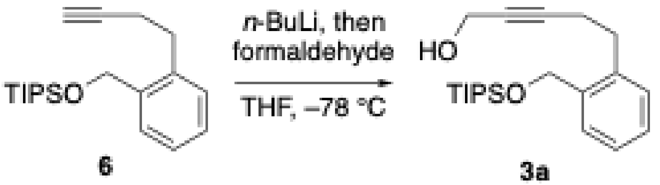

To a solution of **6** (309 mg, 0.976 mmol) in THF (10 mL) was added *n*-BuLi (1.31 M in hexane, 899 μL, 1.17 mmol) at −78 °C, and the mixture was stirred for 10 min at that temperature. After the mixture was warmed to 0 °C and stirred for 20 min, formaldehyde gas (excess, prepared by thermal cracking of solid paraformaldehyde using a heat gun) was bubbled through the mixture at 0 °C, and stirred at that temperature for 30 min. The reaction was quenched with sat. aq. NH_4Cl and extracted with AcOEt. The combined organic phase was washed with brine and dried over Na_2SO_4, filtered and the mixture was concentrated under reduced pressure. The residue was purified by silica gel chromatography (hexane/AcOEt 60:40) to afford 281 mg (83%) of **3a** as a colorless syrup.

^{1}H NMR (300 MHz, $CDCl_3$): δ 7.57-7.43 (m, 1H), 7.30-7.14 (m, 3H), 4.85 (s, 2H), 4.23 (t, *J* = 2.0 Hz, 2H), 2.84 (t, *J* = 7.7 Hz, 2H), 2.52 (tt, *J* = 7.7, 2.0 Hz, 2H), 1.06-1.27 (m, 21H).

^{13}C{^{1}H} NMR (75 MHz, $CDCl_3$): δ 138.96, 137.47, 128.74, 127.08, 127.00, 126.49, 85.86, 78.99, 63.14, 51.39, 31.11, 20.09, 18.09, 12.05.

HRMS (FAB, matrix: 3-nitrobenzyl alcohol, positive): Exact mass calcd. for $C_{21}H_{33}O_2Si$ $[M–H]^+$ requires *m/z*: 345.2250, found *m/z*: 345.2250.

IR (ATR, cm^{-1}): 3330, 2942, 2890, 2865, 2231, 1692, 1462, 1117, 1065.

(*Z*)-3-Iodo-5-(2-(((triisopropylsilyl)oxy)methyl)phenyl)pent-2-en-1-ol (7)

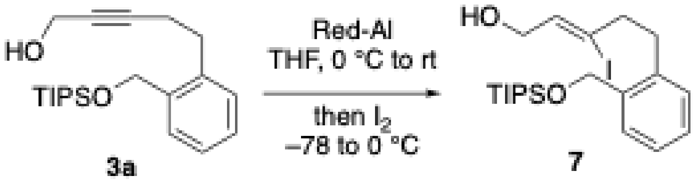

To a solution of **3a** (3.68 g, 10.6 mmol) in THF (100 mL) was added Red-Al (1.44 M in toluene, 11.1 mL, 15.9 mmol) at 0 °C, and the mixture was stirred at ambient temperature for 3 h. AcOEt (1.04 mL, 10.6 mmol) was added to the mixture at 0 °C to quench the excess Red-Al. After the mixture was cooled to −78 °C, a solution of I_2 (4.04 g, 15.9 mmol) in THF (15 mL) was added to the mixture and stirred at that temperature for 30 min, then warmed to 0 °C and stirred for additional 2 h. The reaction was quenched with sat. aq. NH_4Cl and extracted with AcOEt. The combined organic phase was washed with brine and dried over Na_2SO_4, filtered and the filtrate was concentrated under reduced pressure. The residue was purified by silica gel chromatography (hexane/AcOEt 80:20) to afford 4.06 g (81%) of **7** as a colorless syrup.

^{1}H NMR (300 MHz, $CDCl_3$): δ 7.52 (dd, *J* = 6.6, 2.3 Hz, 1H), 7.24-7.13 (m, 3H), 5.78 (td, *J* = 5.9, 1.0 Hz, 1H), 4.86 (s, 2H), 4.18 (dd, *J* = 5.9, 5.9 Hz, 2H), 2.86-2.73 (m, 4H), 1.25-1.09 (m, 21H).

^{13}C{^{1}H} NMR (75 MHz, $CDCl_3$): δ 139.06, 136.97, 134.23, 129.09, 126.99, 126.91, 126.45, 109.11, 67.27, 63.02, 46.54, 32.09, 18.15, 12.05.

HRMS (FAB, matrix: 3-nitrobenzyl alcohol, positive): Exact mass calcd. for $C_{21}H_{34}O_2SiI$ $[M–H]^+$ requires *m/z*: 473.1373, found *m/z*: 473.1373.

IR (ATR, cm^{-1}): 3313, 2941, 2864, 1645, 1460, 1119, 1065, 1013.

(*Z*)-((2-(5-Chloro-3-iodopent-3-en-1-yl)benzyl)oxy)triisopropylsilane (10)

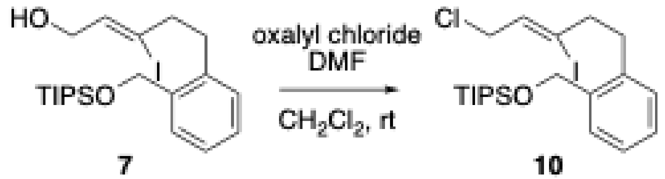

7 10

To a solution of DMF (543 μL, 7.02 mmol) in CH_2Cl_2 (10 mL) was added oxalyl chloride (451 μL, 5.26 mmol) at 0 °C, and the mixture was stirred at that temperature for 20 min. After the addition of a solution of **7** (1.66 g, 3.51 mmol) in CH_2Cl_2 (10 mL), the mixture was stirred at ambient temperature for 1.5 h. The reaction was quenched with sat. aq. $NaHCO_3$ and extracted with AcOEt. The combined organic phase was washed with brine and dried over Na_2SO_4, filtered and the filtrate was concentrated under reduced pressure. The residue was purified by silica gel chromatography (hexane/AcOEt 98:2) to afford 1.49 g (86%) of **10** as a colorless syrup.

^{1}H NMR (300 MHz, $CDCl_3$): δ 7.54 (dd, *J* = 6.5, 2.5 Hz, 1H), 7.30-7.22 (m, 2H), 7.17 (dd, *J* = 6.7, 2.5 Hz, 1H), 5.77 (t, *J* = 7.1 Hz, 1H), 4.90 (s, 2H), 4.14 (d, *J* = 7.1 Hz, 2H), 2.97-2.78 (m, 4H), 1.34-1.13 (m, 21H).

$^{13}C\{^1H\}$ NMR (75 MHz, $CDCl_3$): δ 139.00, 136.92, 131.15, 129.12, 127.12, 127.10, 126.48, 113.07, 63.14, 47.87, 46.58, 32.06, 18.13, 12.05.

HRMS (FAB, matrix: 3-nitrobenzyl alcohol, positive): Exact mass calcd. for $C_{21}H_{33}ClOSiI$ $[M–H]^+$ requires *m/z*: 491.1034, found *m/z*: 491.1036.

IR (ATR, cm^{-1}): 3462, 3017, 2941, 2864, 2340, 1739, 1461, 1366, 1217, 1063.

(*Z*)-(2-(5-Chloro-3-iodopent-3-en-1-yl)phenyl)methanol (2a)

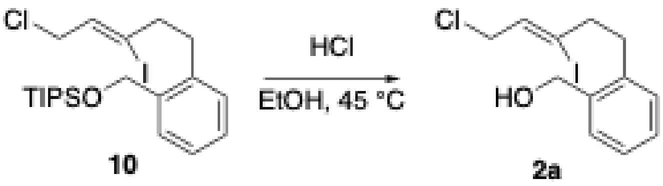

To a solution of **10** (1.49 g, 3.02 mmol) in EtOH (36 mL) was added conc. HCl (1.8 g) at 0 °C, and the mixture was stirred at 45 °C for 5 h. The mixture was concentrated under reduced pressure. The residue was purified by silica gel chromatography (hexane/AcOEt 70:30) to afford 878 mg (86%) of **2a** as a colorless syrup.

^{1}H NMR (300 MHz, $CDCl_3$): δ 7.37 (dd, J = 6.5, 2.6 Hz, 1H), 7.30-7.18 (m, 3H), 5.74 (tt, J = 7.1, 1.2 Hz, 1H), 4.74 (s, 2H), 4.10 (d, J = 7.1, 2H), 2.96-2.91 (m, 2H), 2.87-2.78 (m, 2H), 1.60 (s, 1H).

^{13}C{^{1}H} NMR (75 MHz, $CDCl_3$): δ 138.36, 138.34, 131.28, 129.81, 128.62, 128.19, 126.77, 112.92, 63.30, 47.86, 47.00, 32.21.

HRMS (FAB, matrix: 3-nitrobenzyl alcohol, positive): Exact mass calcd. for $C_{12}H_{13}ClOI$ $[M–H]^+$ requires *m/z*: 334.9700, found *m/z*: 334.9702.

IR (ATR, cm^{-1}): 3332, 3016, 2969, 2944, 1738, 1436, 1365, 1217, 1043.

6-Iodo-oxa[7]orthocyclophene (1ad)

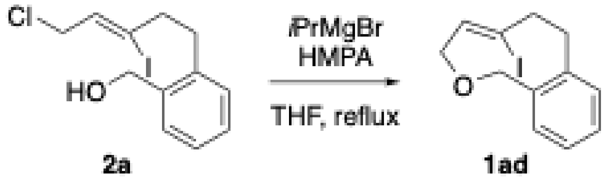

To a solution of **2a** (219 mg ,0.638 mmol) in THF (65 mL) was added HMPA (444 µL, 2.55 mmol) and iPrMgBr (0.655 M, 1.02 mL, 0.669 mmol) at 0 °C and the mixture was stirred under reflux conditions for 4.5 h. The reaction was quenched with MeOH (2 mL) and the mixture was concentrated under reduced pressure. After the addition of sat. aq. $NaHCO_3$, the mixture was extracted with hexane. The combined organic phase was washed with brine and dried over Na_2SO_4, filtered and the mixture was concentrated under reduced pressure. The residue was purified by silica gel chromatography (hexane to hexane/Et_2O 96:4) to afford 152 mg (79%) of **1ad** as a colorless syrup.

^{1}H NMR (300 MHz, $CDCl_3$): δ 7.45-7.33 (m, 1H), 7.29-7.20 (m, 2H), 7.19-7.10 (m, 1H), 4.87 (dd, J = 10.3, 4.4 Hz, 1H), 4.43 (d, J = 12.9 Hz, 1H), 4.40 (dd, J = 10.7, 4.4 Hz, 1H), 4.24 (dd, J = 10.7, 10.3 Hz, 1H), 4.1 (d, J = 12.9 Hz, 1H), 3.06 (ddd, J = 12.6, 5.5, 1.9 Hz, 1H), 2.94 (ddd, J = 13.6, 12.9, 1.6 Hz, 1H), 2.77 (ddd, J = 13.6, 5.5, 1.6 Hz, 1H), 2.60 (ddd, J = 12.9, 12.6, 1.9 Hz, 1H).

^{13}C{^{1}H} NMR (75 MHz, $CDCl_3$): δ 143.41, 136.70, 133.70, 131.81, 131.60, 128.13, 126.93, 117.43, 73.68, 65.50, 48.78, 34.17.

HRMS (EI, positive): Exact mass calcd. for $C_{12}H_{13}OI$ $[M]^+$ requires m/z: 300.0011, found m/z: 300.0012.

IR (ATR, cm^{-1}): 3015, 2963, 2878, 1710, 1627, 1461, 1261, 1216, 1097, 1021.

Analytical HPLC conditions: column: CHIRALCEL OJ-H (4.6 mm × 250 mm), eluent: hexane/iPrOH 95:5, flow rate: 0.5 mL/min, detection: UV 220 nm, temperature: 25 °C, retention time: t_1 = 9.7 min for (R)-**1ad**, t_2 = 12.0 min for (S)-**1ad**.

Optical rotation value: $[\alpha]_D^{23}$ = –241.8 (c 0.96, $CHCl_3$) for (S)-**1ad** (>99% ep).

6-Methyl-oxa[7]orthocyclophene (1ab)

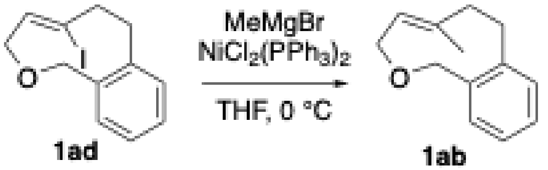

To a solution of **1ad** (60.1 mg, 0.200 mmol) in THF (5 mL) was added $NiCl_2(PPh_3)_2$ (13.1 mg, 0.0200 mmol) and MeMgBr (3.0 M, 567 μL, 0.601 mmol) at 0 °C, and the mixture was stirred at that temperature for 30 min. The reaction was quenched with sat. aq. $NaHCO_3$ and extracted with hexane. The combined organic phase was washed with brine and dried over Na_2SO_4, filtered and the mixture was concentrated under reduced pressure. The residue was purified by silica gel chromatography (hexane to hexane/AcOEt 96:4) to afford 29.8 mg (79%) of **1ab** as a colorless syrup.

^{1}H NMR (300 MHz, $CDCl_3$): δ 7.47-7.37 (m, 1H), 7.35-7.24 (m, 2H), 7.24-7.12 (m, 1H), 4.75 (dd, J = 12.8, 4.3 Hz, 1H), 4.43 (d, J = 12.8 Hz, 1H), 4.39 (dd, J = 12.8, 10.5 Hz, 1H), 4.15 (dd, J = 10.5, 4.3 Hz, 1H), 3.86 (d, J = 12.8 Hz, 1H), 2.88 (ddd, J = 13.2, 5.4, 2.1 Hz, 1H), 2.72 (ddd, J = 13.2, 12.1, 1.8 Hz, 1H), 2.52 (ddd, J = 11.9, 5.4, 1.8 Hz, 1H), 2.02 (s, 3H), 2.01 (ddd, J = 12.1, 11.9, 2.1 Hz, 1H).

^{13}C{^{1}H} NMR (75 MHz, $CDCl_3$): δ 143.92, 141.62, 138.40, 131.33, 127.72, 126.70, 124.32, 66.05, 64.32, 40.79, 33.85, 17.92. (Two aromatic carbons were observed as one peak due to overlapping)

HRMS (EI, positive): Exact mass calcd. for $C_{13}H_{16}O$ $[M]^+$ requires m/z: 188.1201, found m/z: 188.1199.

IR (ATR, cm^{-1}): 3054, 3014, 2930, 2873, 1459, 1036, 1016.

Analytical HPLC conditions: CHIRALCEL OJ-H (4.6 mm x 250 mm), eluent: hexane/*i*PrOH = 95:5, flow rate: 0.5 mL/min, detection: UV 220 nm, temperature: 25 °C, retention time: t_1 = 9.9 min for (*R*)-**1ab**, t_2 = 11.8 min for (*S*)-**1ab**.

Optical rotation value: $[\alpha]_D^{23}$ = –161.2 (c 1.02, $CHCl_3$) for (*S*)-**1ab** (>99% ep).

6-Phenyl-oxa[7]orthocyclophene (1ac)

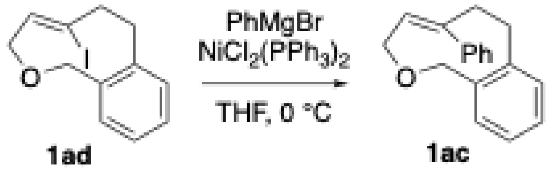

To a solution of **1ad** (7.60 mg, 0.0293 mmol) in THF (5 mL) was added $NiCl_2(PPh_3)_2$ (1.92 mg, 0.00293 mmol) and PhMgBr (1.0 M, 147 µL, 0.147 mmol) at 0 °C, and the mixture was stirred at that temperature for 15 min. The reaction was quenched with sat. aq. $NaHCO_3$ and extracted with hexane. The combined organic phase was washed with brine and dried over Na_2SO_4, filtered and the mixture was concentrated under reduced pressure. The residue was purified by PTLC (hexane/Et_2O 90:10) to afford 7.3 mg (99%) of **1ac** as colorless crystals.

^{1}H NMR (300 MHz, $CDCl_3$): δ 7.48-7.26 (m, 6H), 7.31-7.21 (m, 2H), 7.25-7.16 (m, 1H), 5.13 (dd, *J* = 11.2, 3.9 Hz, 1H), 4.36 (d, *J* = 12.6 Hz, 1H), 4.29-4.43 (m, 2H), 3.93 (d, *J* = 12.6 Hz, 1H), 3.19-3.09 (m, 2H), 3.02-2.96 (m, 1H), 2.37-2.28 (m, 1H).

^{13}C{^{1}H} NMR (150 MHz, $CDCl_3$): δ 146.13, 143.59, 139.83, 138.01, 131.77, 131.46, 128.61, 128.36, 128.23, 128.14, 127.13, 127.07, 69.19, 65.27, 39.19, 36.73.

HRMS (EI, positive): Exact mass calcd. for $C_{18}H_{18}O$ $[M]^+$ requires *m/z*: 250.1358, found *m/z*: 250.1358.

IR (ATR, cm^{-1}): 3055, 3020, 2948, 2872, 1492, 1456, 1447, 1024, 769, 765, 701.

Analytical HPLC conditions: column: CHIRALCEL OJ-H (4.6 mm x 250 mm), eluent: hexane/*i*PrOH = 95:5, flow rate: 1.0 mL/min, detection: UV 220 nm, temperature: 25 °C, retention time: t_1 = 6.7 min, t_2 = 7.4 min.

m.p. 128.5-129.0 °C

Single crystal X-ray crystallography:

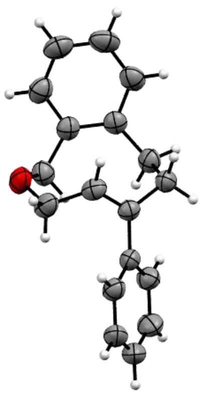

Crystal data (CCDC 2513894): $C_{18}H_{18}O$, monoclinic, $P2_1/c$, a = 8.6010(2) Å, b = 20.0405(4) Å, c = 7.9500(2) Å, β = 101.172(2)° , V = 1344.36(5) Å^3, T = 100(2) K, Z = 4, radiation type: CuK_α, 13669 reflections measured, 2875 unique (Rint = 0.0297) which were used in all calculations. The final wR_2 was 0.1236 (all data) and R_1 was 0.0467.

ORTEP drawing of **1ac** (ellipsoid set at 50% probability level)

(5*R*,6*R*)-6-Methyl-oxa[7]orthocyclophene oxide ((5*R*,6*R*)-8)

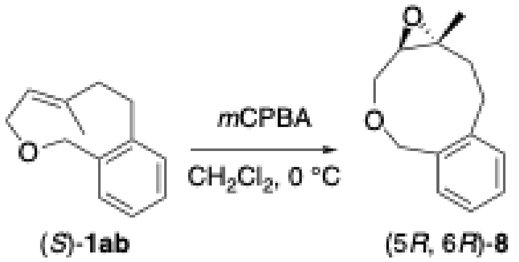

To a solution of (*S*)-**1ab** (7.44 mg, 0.0395 mmol) in CH_2Cl_2 (1 mL) was added *m*-CPBA (contains ca. 30% water, 31.0 mg, 0.118 mmol) at 0 °C and the mixture was stirred at that temperature for 30 min. The reaction was quenched with sat. aq. $Na_2S_2O_3$ and extracted with CH_2Cl_2. The combined organic phase was dried over Na_2SO_4, filtered and the mixture was concentrated under reduced pressure. The residue was purified by silica gel chromatography (hexane to hexane/AcOEt 90:10) to afford 4.9 mg (61%) of (5*R*,6*R*)-**8** as a colorless syrup.

^{1}H NMR (400 MHz, $CDCl_3$): δ 7.44-7.38 (m, 1H), 7.29-7.19 (m, 2H), 7.13-7.07 (m, 1H), 4.64 (d, *J* = 13.0 Hz, 1H), 4.38 (d, *J* = 13.0 Hz, 1H), 4.08 (dd, *J* = 11.0, 2.7 Hz, 1H), 3.53 (dd, *J* = 11.0, 11.0 Hz, 1H), 2.92 (ddd, *J* = 14.3, 13.8, 1.8 Hz, 1H), 2.79 (ddd, *J* = 14.3, 6.1, 2.1 Hz, 1H), 2.54 (dd, *J* = 11.0, 2.7 Hz, 1H), 2.38 (ddd, *J* = 12.6, 6.1, 1.8 Hz, 1H), 1.56 (s, 3H), 1.19 (ddd, *J* = 13.8, 12.6, 2.1 Hz, 1H).

^{13}C{^{1}H} NMR (100 MHz, $CDCl_3$): δ 140.55, 138.19, 131.47, 131.18, 128.49, 127.32, 66.61, 66.25, 59.08, 58.00, 38.50, 30.06, 18.15.

HRMS (EI, positive): Exact mass calcd. for $C_{13}H_{16}O_2$ $[M]^+$ requires *m*/*z*: 204.1150, found *m*/*z*: 204.1151.

IR (ATR, cm^{-1}): 2951, 2882, 2357, 2325, 1471, 1108, 1061, 768.

Analytical HPLC conditions: column: CHIRALPAK IH (4.6 mm × 250 mm), eluent: hexane/iPrOH 90:10, flow rate: 1.0 mL/min, detection: UV 220 nm, temperature: 25 °C, retention time: t_1 = 5.9 min for (5*R,*6*R*)-**8**, t_2 = 16.0 min for (5*S,*6*S*)-**8**

Optical rotation value: $[\alpha]_D^{23}$ = –278.9 (c 0.628, $CHCl_3$) for (5*R*, 6*R*)-**8** (>99% ep).

3. Copies of ^{1}H and ^{13}C NMR spectra

^{1}H NMR chart (300 MHz in $CDCl_3$) of **6**

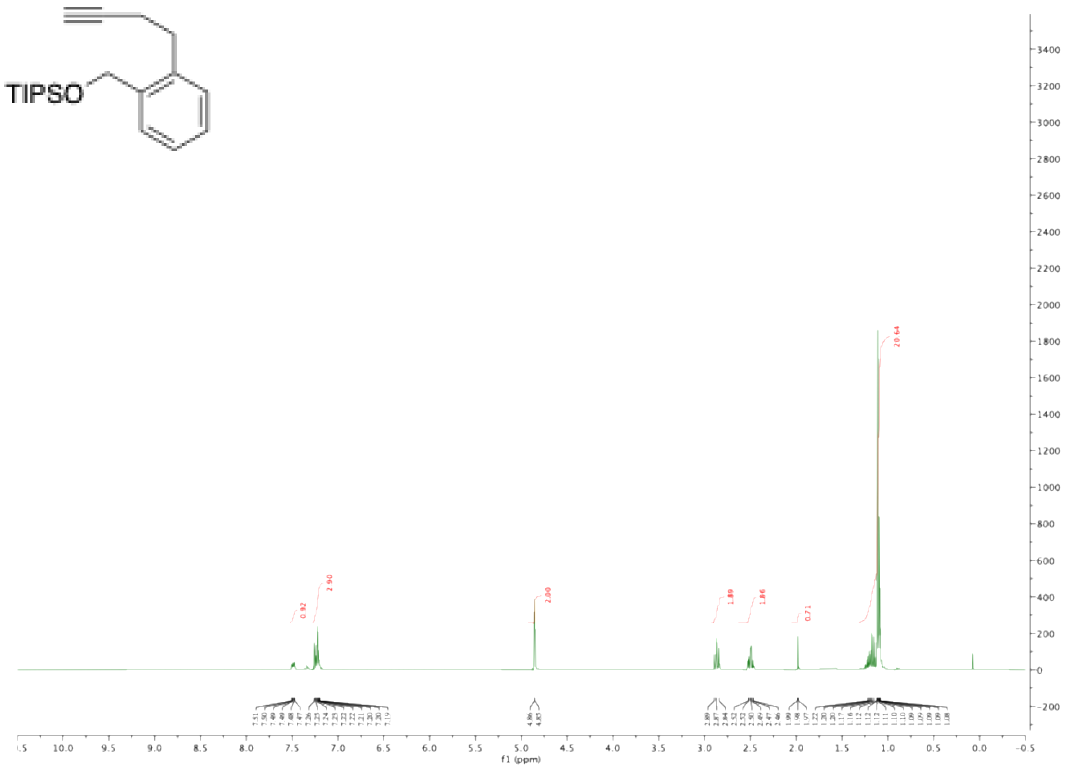

$^{13}C\{^{1}H\}$ NMR chart (75 MHz in $CDCl_3$) of **6**

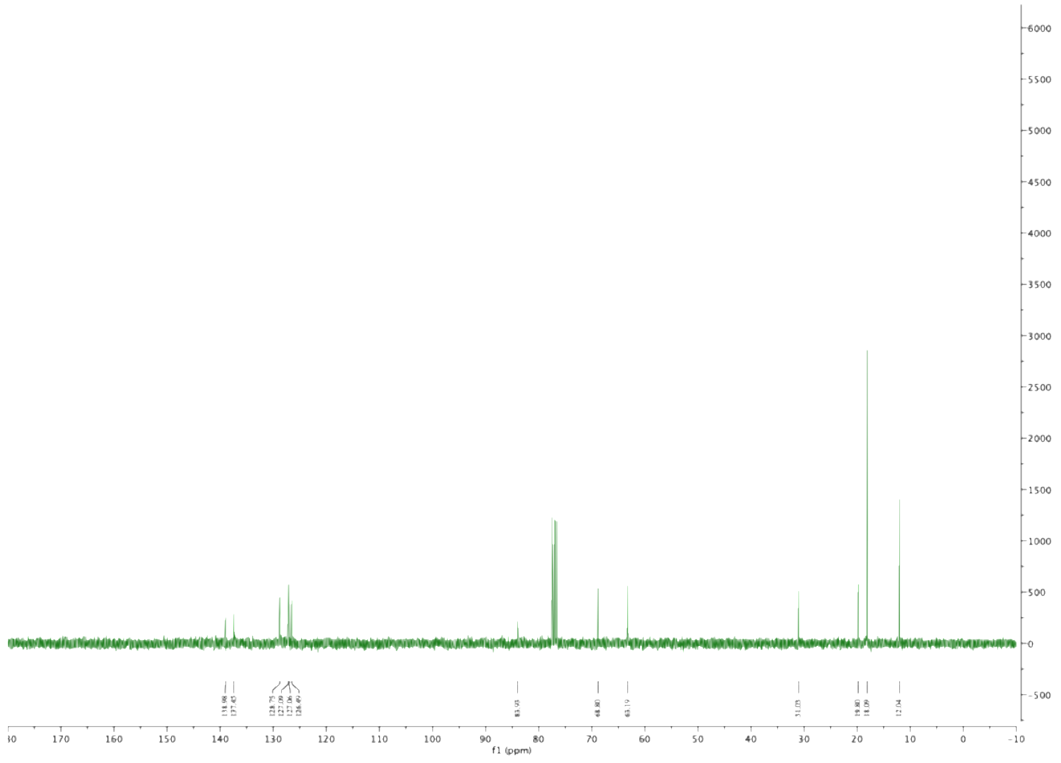

^{1}H NMR chart (300 MHz in $CDCl_3$) of **3a**

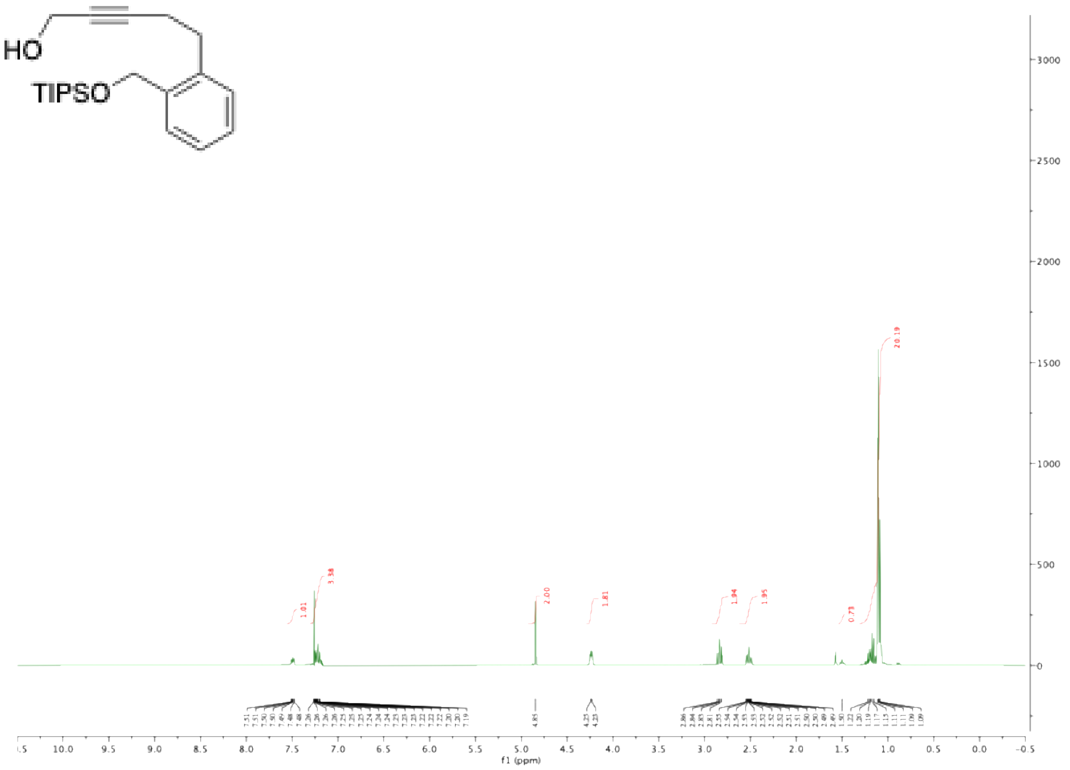

^{13}C{^{1}H} NMR chart (75 MHz in $CDCl_3$) of **3a**

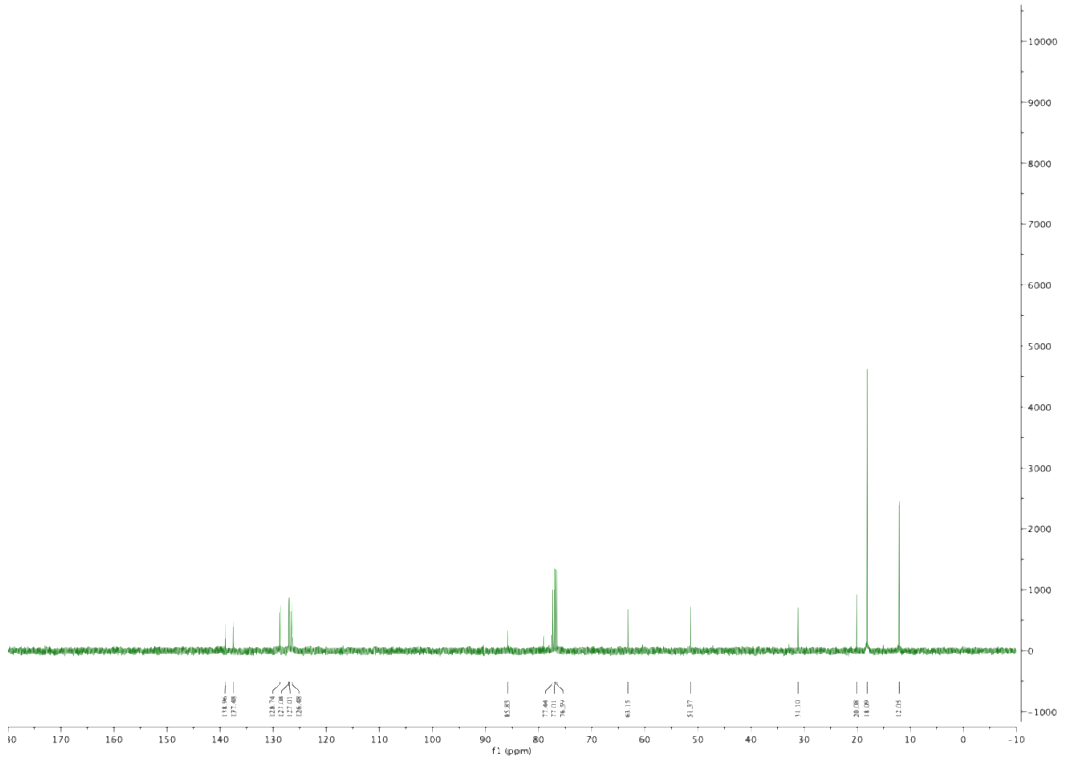

^{1}H NMR chart (300 MHz in $CDCl_3$) of **7**

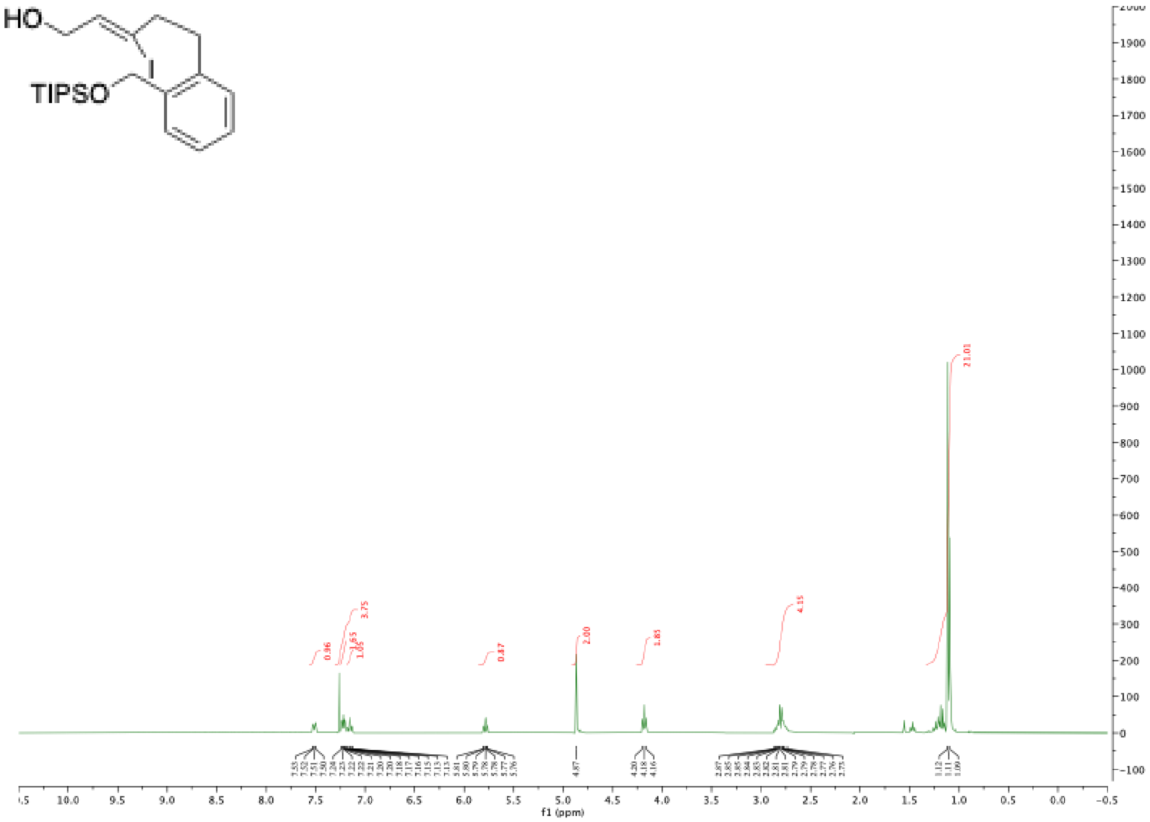

^{13}C{^{1}H} NMR chart (75 MHz in $CDCl_3$) of **7**

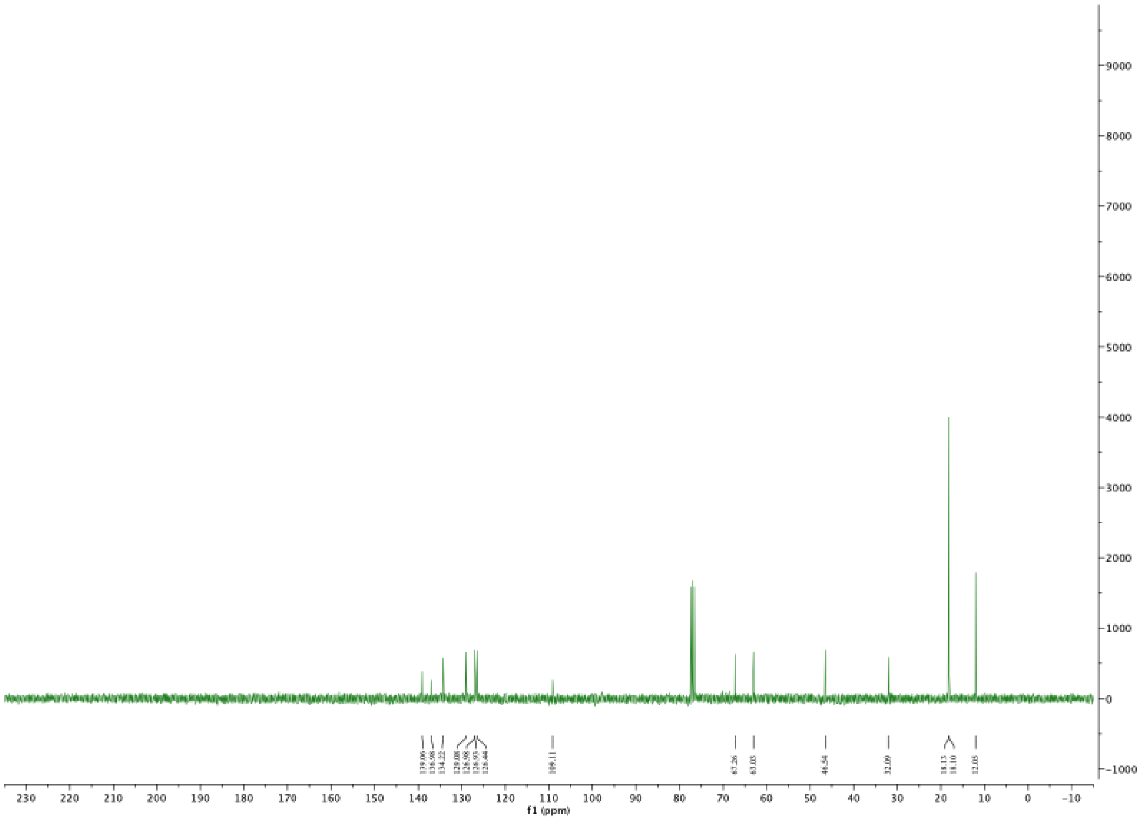

^{1}H NMR (300 MHz in $CDCl_3$) chart of **10**

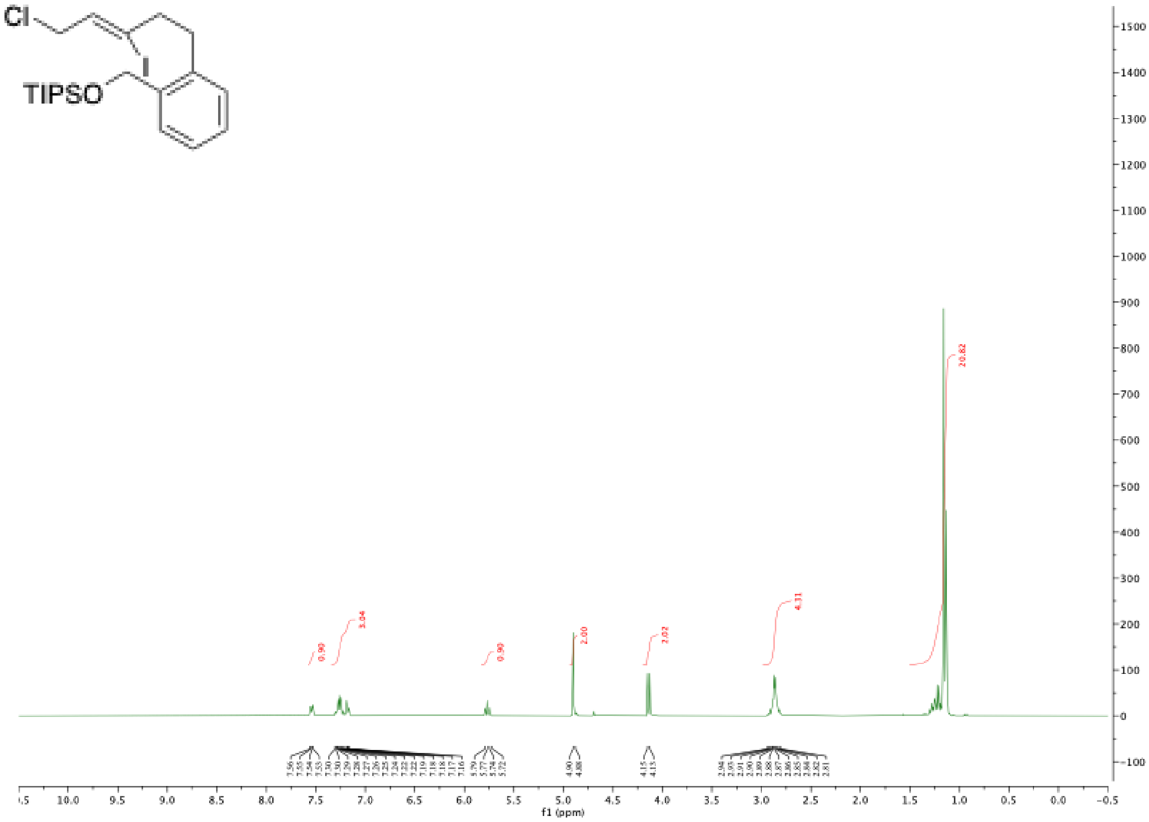

$^{13}C\{^{1}H\}$ NMR chart (75 MHz in $CDCl_3$) of **10**

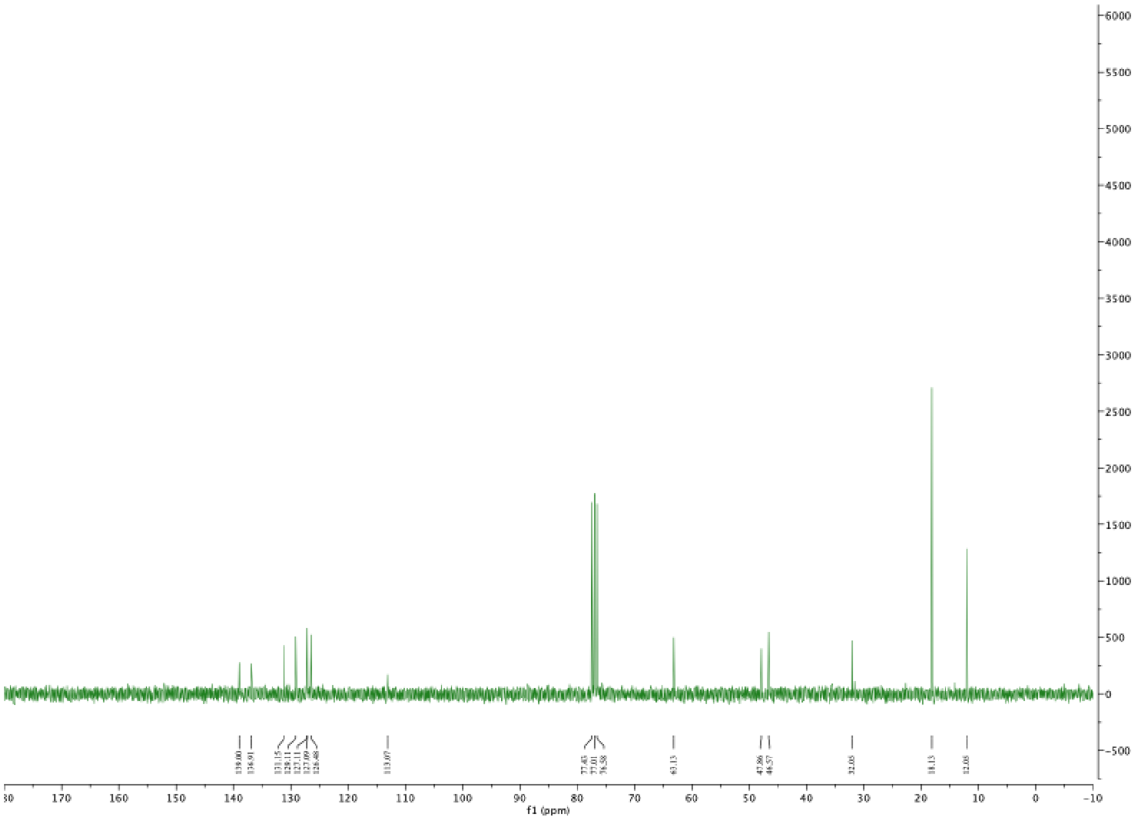

^{1}H NMR chart (300 MHz in $CDCl_3$) of **2a**

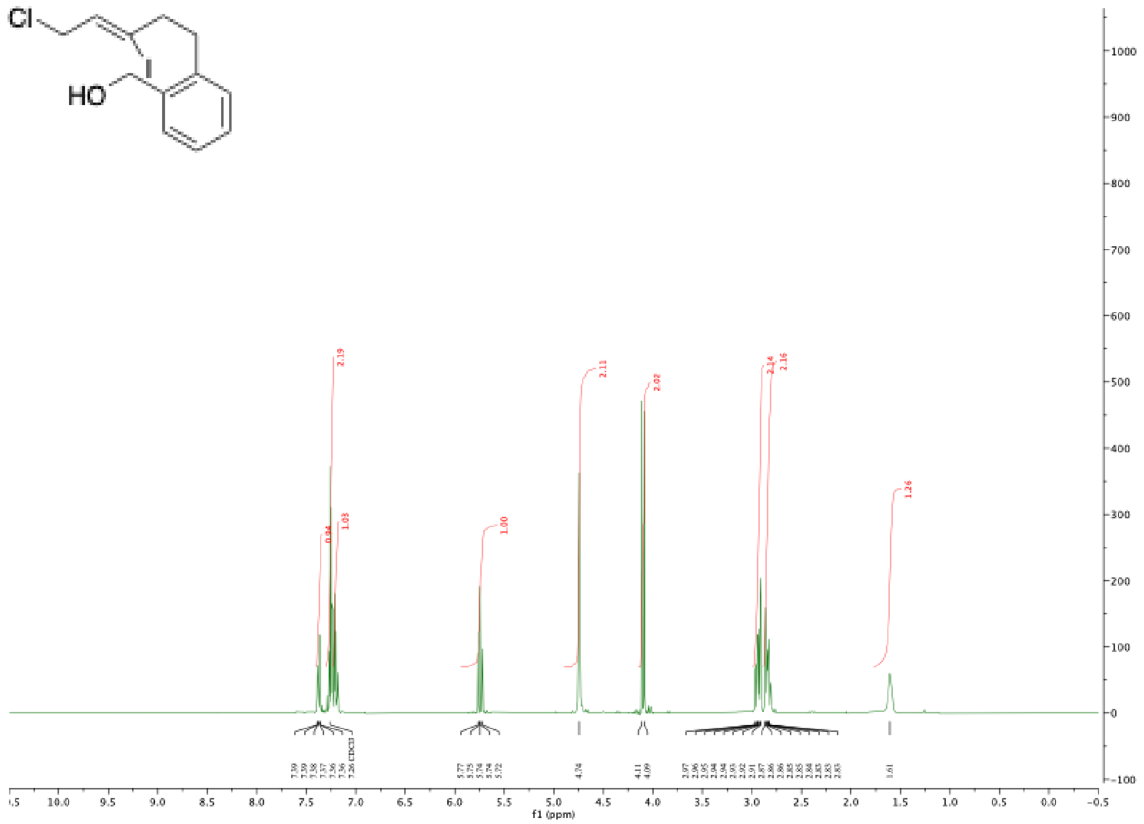

^{13}C{^{1}H} NMR chart (75 MHz in $CDCl_3$) of **2a**

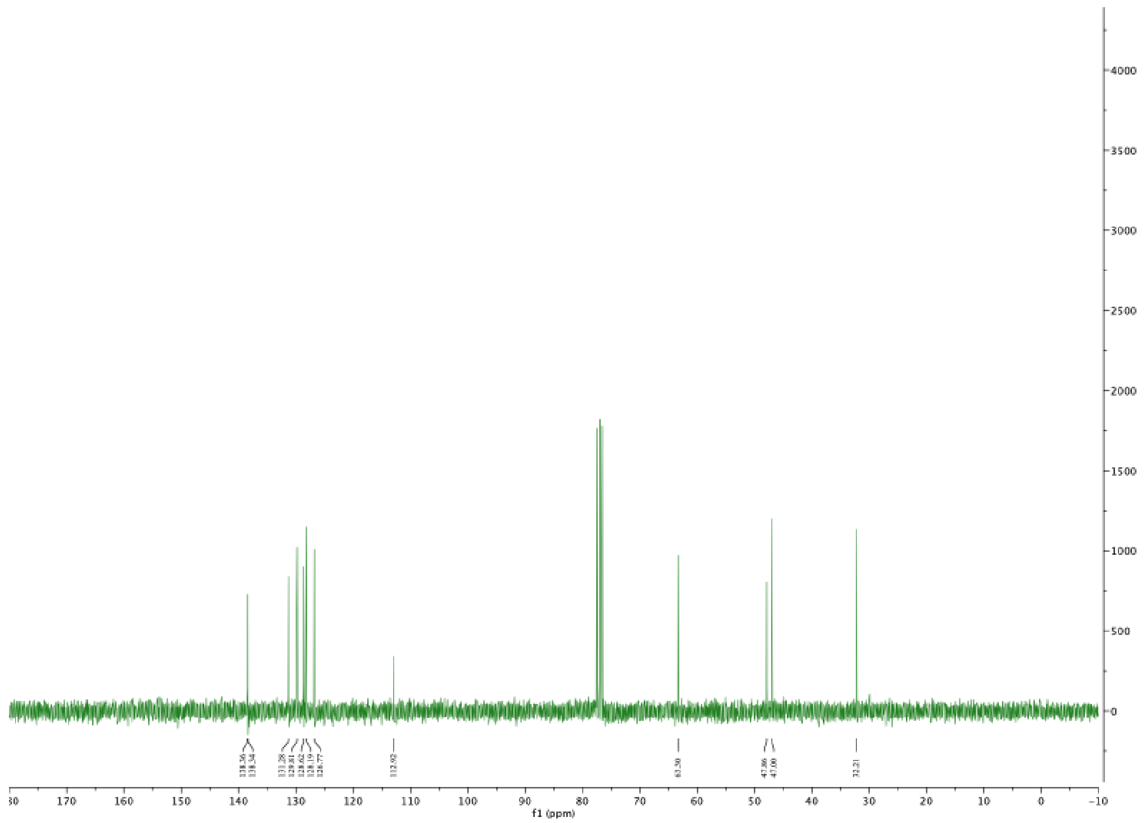

^{1}H NMR chart (300 MHz in $CDCl_3$) of **1ad**

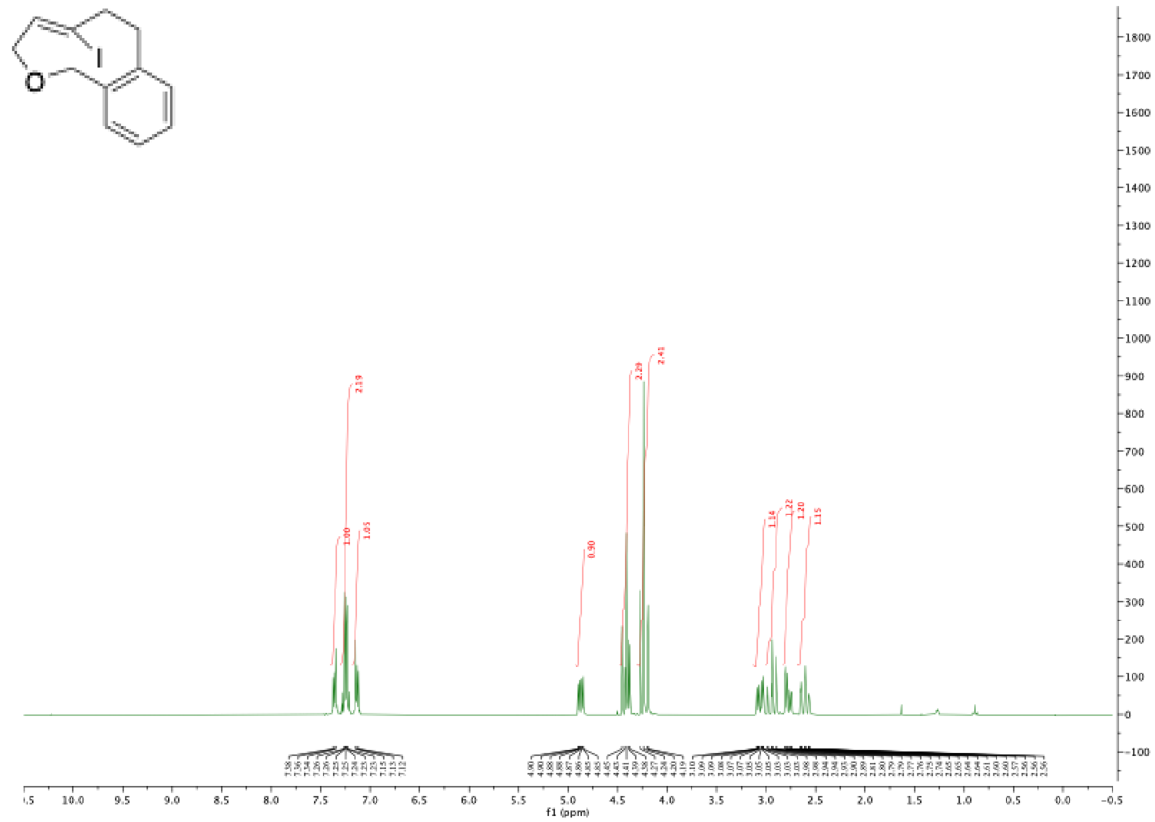

^{13}C{^{1}H} NMR chart (75 MHz in $CDCl_3$) of **1ad**

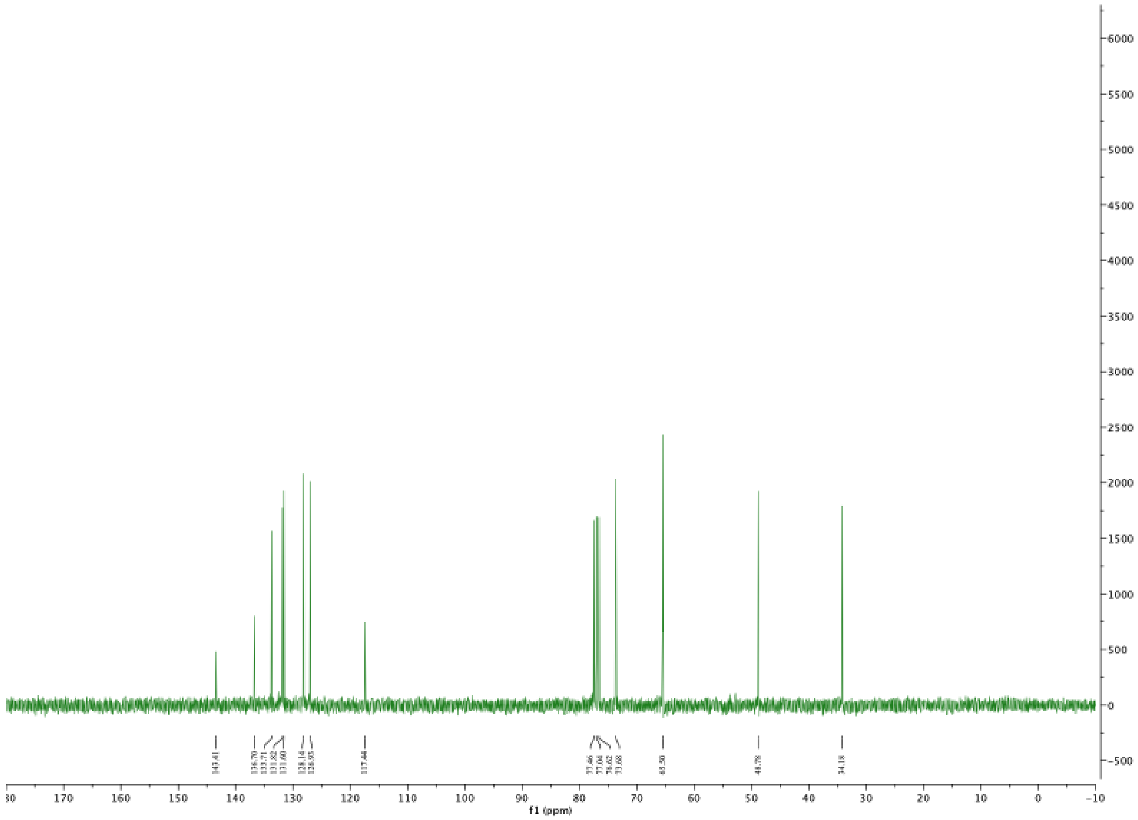

^{1}H NMR chart (300 MHz in $CDCl_3$) of **1ab**

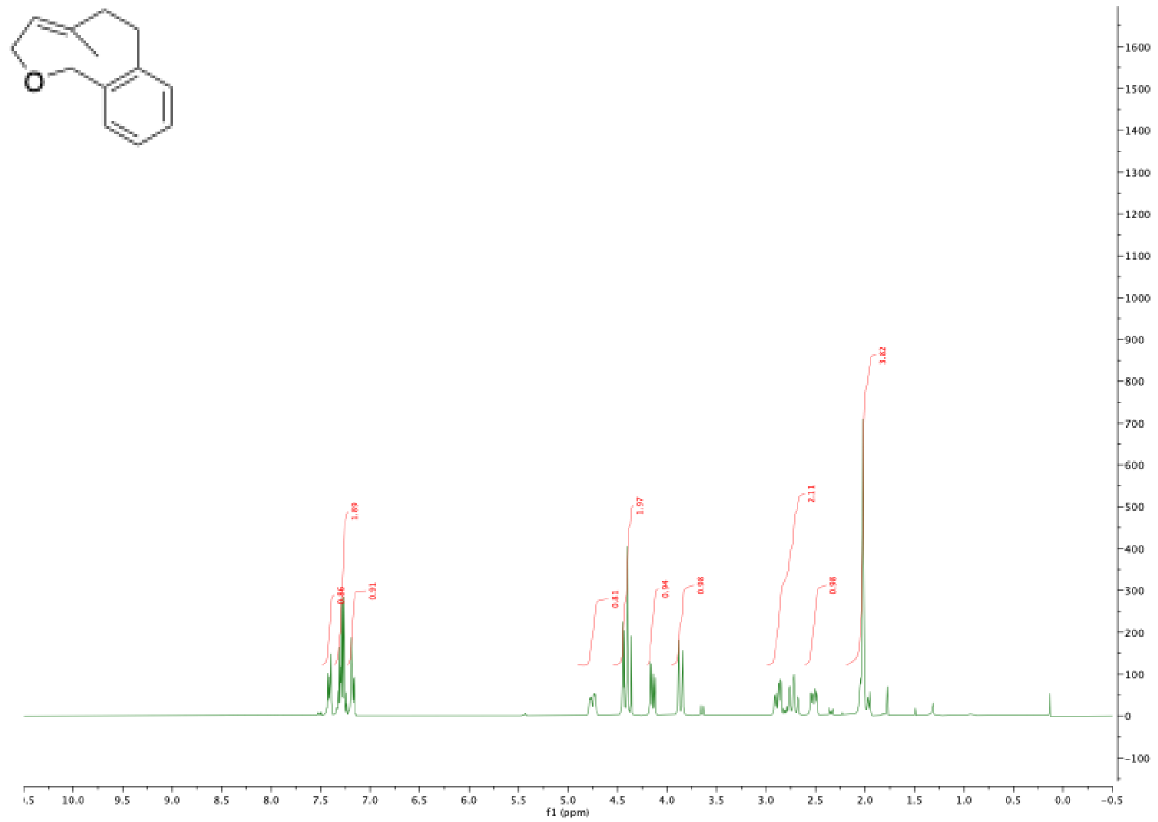

^{13}C{^{1}H} NMR chart (150 MHz in $CDCl_3$) of **1ab**

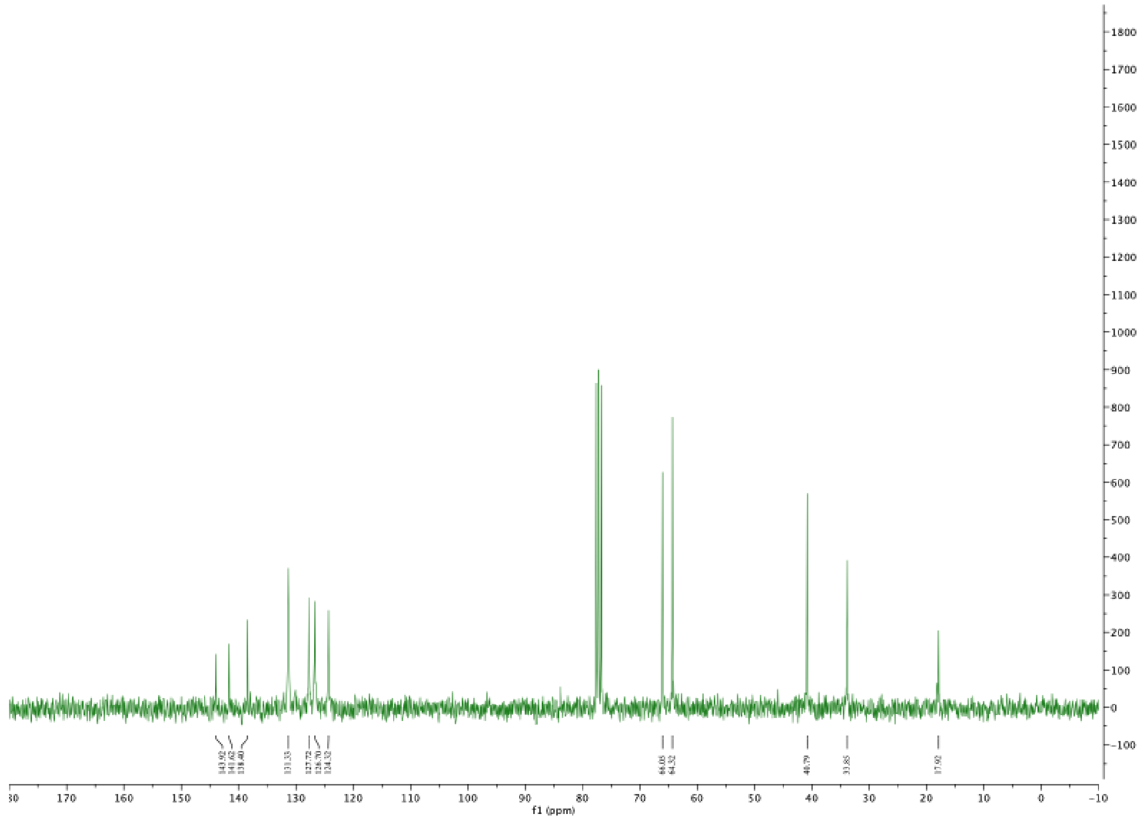

^{1}H NMR chart (300 MHz in $CDCl_3$) of **1ac**

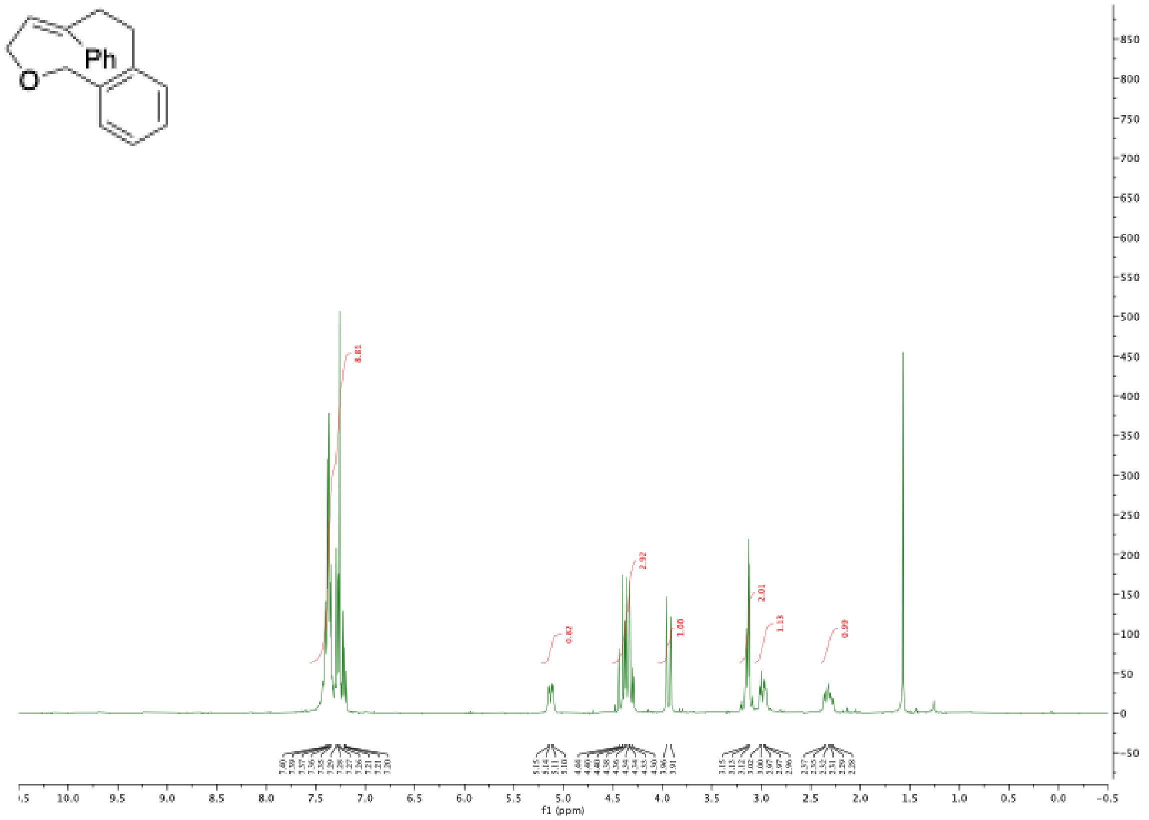

$^{13}C\{^{1}H\}$ NMR chart (150 MHz in $CDCl_3$) of **1ac**

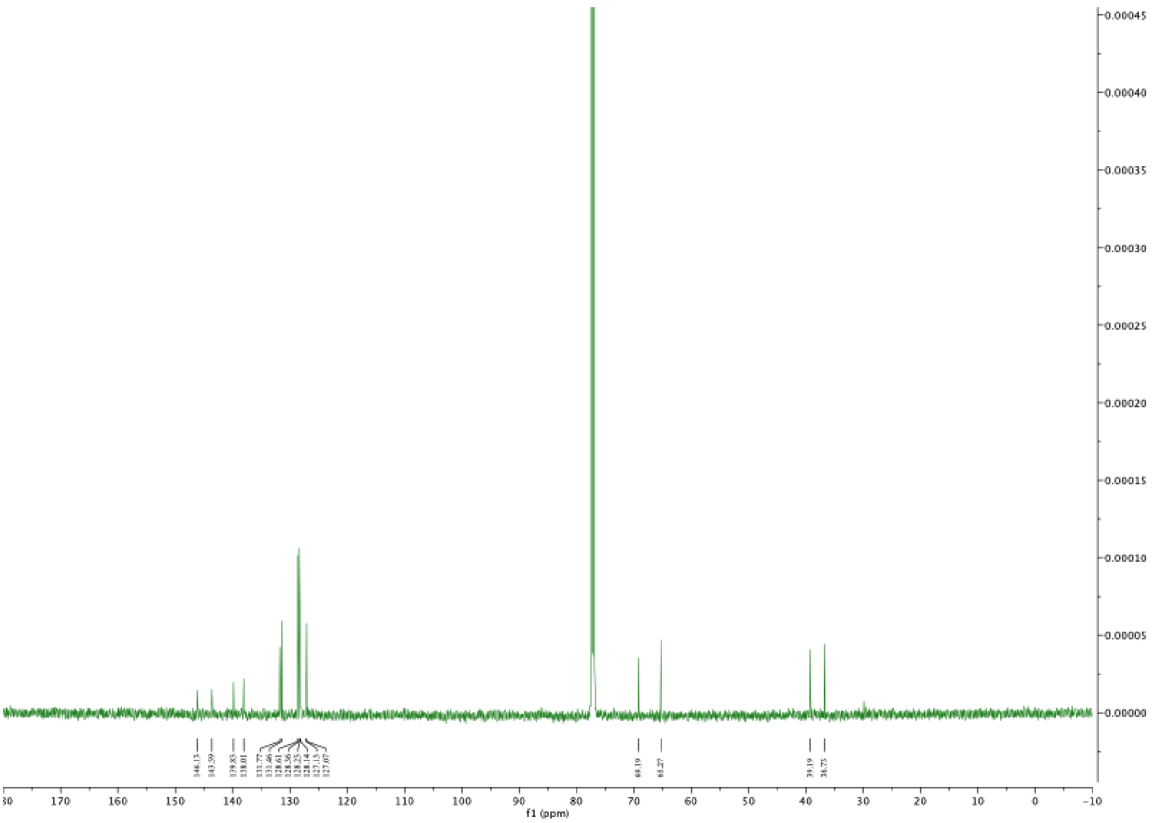

^{1}H NMR chart (400 MHz in $CDCl_3$) of **8**

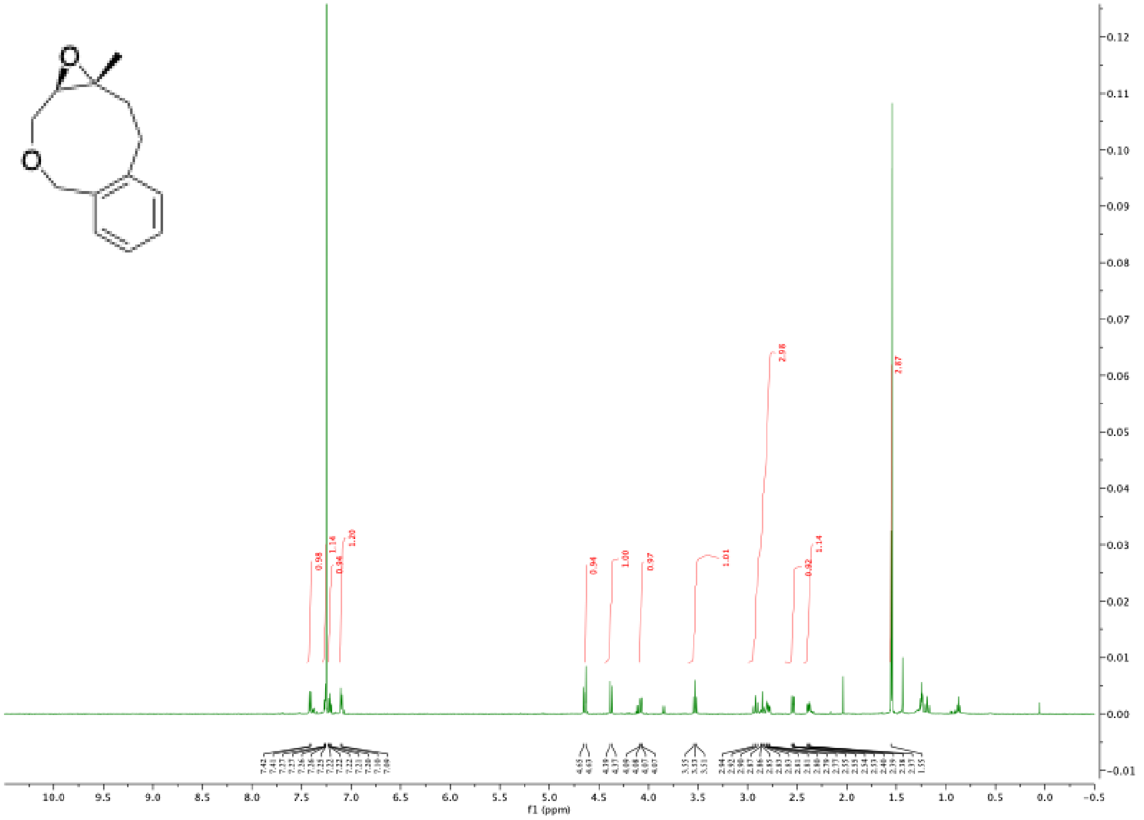

^{13}C{^{1}H} NMR chart (100 MHz in $CDCl_3$) of **8**

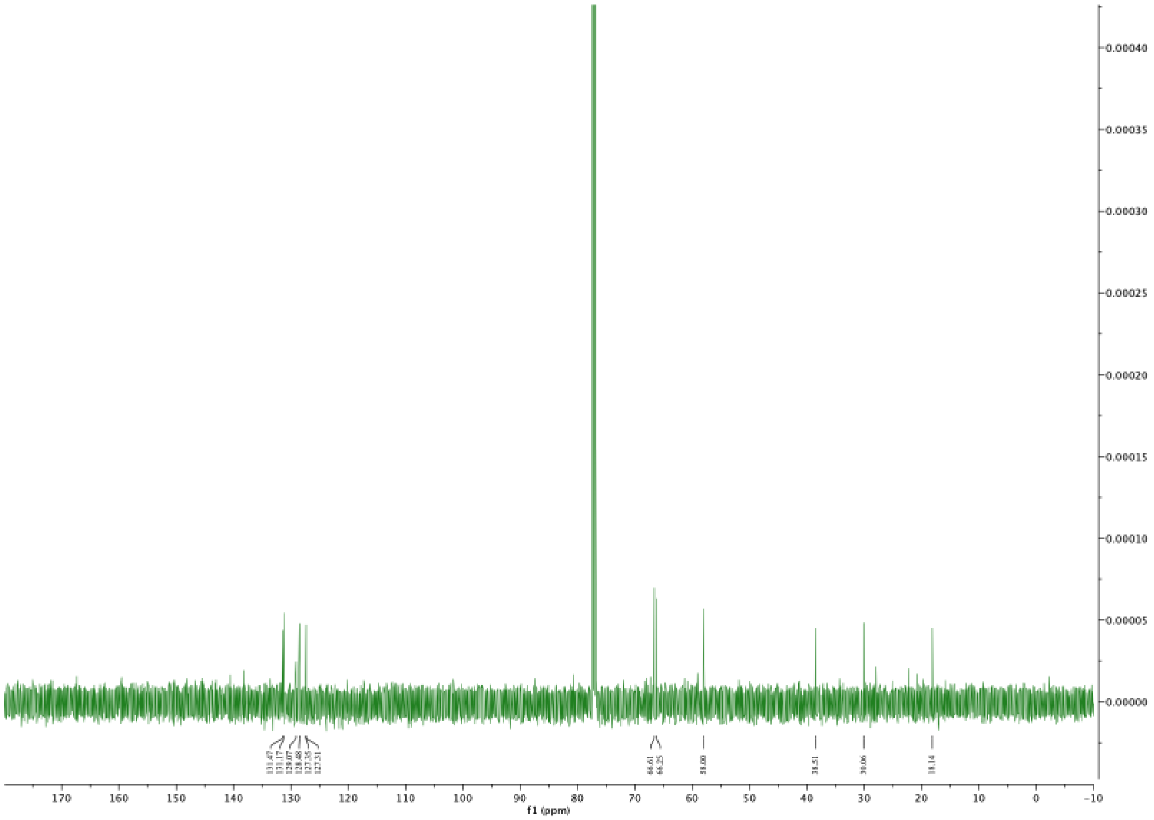

4. DFT calculations

All DFT calculations were performed at the B3LYP level using the 6-311G(2d,2p) basis set for C, H, and O atoms and the SDD basis set with the corresponding effective core potential for iodine by use of the Gaussian 16 program at the computer facilities at Research Institute for Information Technology, Kyushu University. Gaussian 16: Revision C.01, M. J. Frisch, *et al*, Gaussian, Inc., Wallingford CT, 2019. After optimization of the geometries, TD-DFT calculations were performed with *N*states = 30.

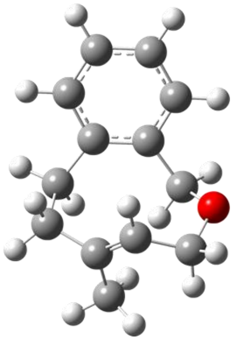

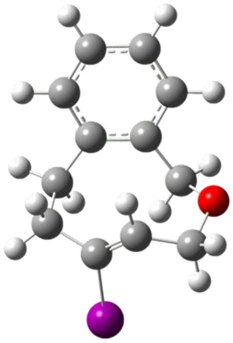

O1	-0.7675	-2.2554	-0.1030
C2	-1.8560	-1.7718	0.7215
C3	-1.5854	-0.3602	1.1178
C4	-2.0471	0.7224	0.4830
C5	-1.2025	1.9676	0.4626
C6	-0.1589	1.8256	-0.6952
C7	0.9823	0.8705	-0.3937
C8	0.9374	-0.5308	-0.5458
C9	-0.2427	-1.3393	-1.0738
C10	2.0609	-1.2844	-0.1866
C11	3.2121	-0.6999	0.3146
C12	3.2625	0.6813	0.4612
C13	2.1595	1.4423	0.1080
H14	-1.8768	-2.4566	1.5681
H15	-2.7960	-1.8780	0.1751
H16	-0.7266	-0.2362	1.7680
H17	-0.6703	2.1064	1.4037
H18	-1.8033	2.8620	0.2851
H19	0.2685	2.8096	-0.8919
H20	-0.6838	1.5382	-1.6069
H21	0.1132	-1.9817	-1.8806
H22	-1.0238	-0.7037	-1.4868
H23	2.0107	-2.3601	-0.2949
H24	4.0621	-1.3132	0.5819
H25	4.1536	1.1623	0.8418
H26	2.2042	2.5187	0.2207
C27	-3.2508	0.7489	-0.4216
H28	-3.7667	-0.2053	-0.4815
H29	-3.9652	1.4898	-0.0529
H30	-2.9863	1.0577	-1.4369

O1	-0.7411	2.4107	-0.0170
C2	0.3260	2.1299	0.9101
C3	0.1943	0.7175	1.3705
C4	0.8257	-0.3489	0.9000
C5	0.1896	-1.6982	0.8047
C6	-0.7076	-1.7310	-0.4756
C7	-1.9952	-0.9356	-0.3505
C8	-2.1263	0.4515	-0.5705
C9	-1.0157	1.4008	-1.0046
C10	-3.3777	1.0489	-0.3776
C11	-4.4873	0.3246	0.0245
C12	-4.3633	-1.0432	0.2381
C13	-3.1326	-1.6512	0.0492
H14	0.1911	2.8500	1.7152
H15	1.2946	2.3059	0.4388
H16	-0.7122	0.5082	1.9310
H17	-0.4363	-1.8664	1.6835
H18	0.9221	-2.5007	0.7581
H19	-0.9687	-2.7711	-0.6724
H20	-0.1098	-1.4013	-1.3248
H21	-1.3607	1.9639	-1.8724
H22	-0.1097	0.8759	-1.2979
H23	-3.4653	2.1155	-0.5385
H24	-5.4393	0.8191	0.1630
H25	-5.2178	-1.6325	0.5427
H26	-3.0420	-2.7182	0.2114
I27	2.6869	-0.1854	-0.2021

Kinetic measurements of the racemization

The chiral HPLC measurement of enantiopurity of (*S*)- or (*R*)-**1ad** and **1ab** was carried out after proper time intervals in hexane/2-propanol 95:5 at 25 °C. Plotting ln a (a = |(*S*)–(*R*)| / |(*S*)+(*R*)|) against time, furnished a straight line, afforded the rate constants *k* of **1ab** and **1ad**. The Gibbs free energy of activation ($\Delta G^{\ddagger}$) and half-lives of optical activity ($_{opt}t_{1/2}$) of **1ab** and **1ad** at 25 °C ware calculated using the following equations.

$$\Delta G^{\ddagger} = -RTln\left(\frac{kh}{2k_BT}\right), \qquad {}_{opt}t_{1/2} = \frac{ln(2)}{k}$$

[k_B: Boltzmann constant, *T*: absolute temperature, *h*: Planck constant, *R*: gas constant]

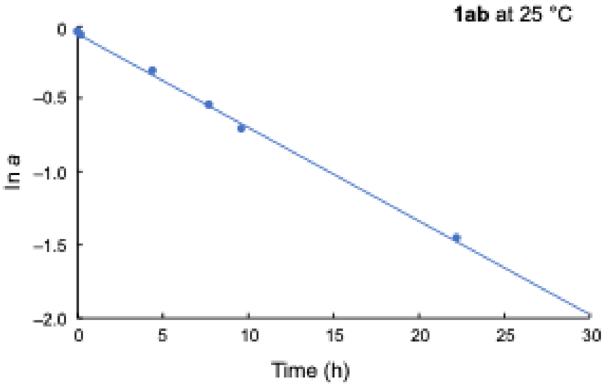

time (h)	%ee	a
0.00	95.8	0.958
0.25	93.2	0.932
4.40	72.9	0.729
7.71	57.6	0.576
9.64	49.1	0.491
22.2	23.2	0.232

T [K]	*k* [$10^{-5}s^{-1}$]	$\Delta G^{\ddagger}$ [kcal/mol]	$_{opt}t_{1/2}$ [h]
298.15	1.779	24.4	10.8

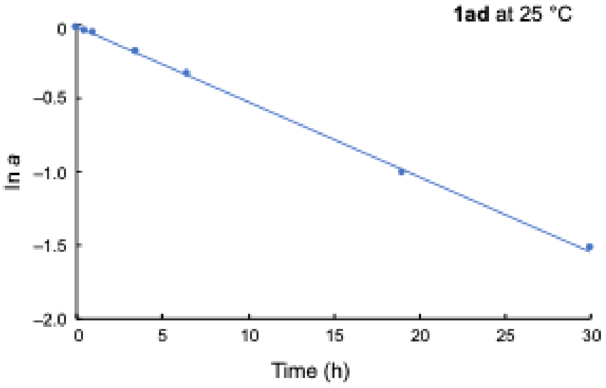

time (h)	%ee	a
0.00	98.5	0.985
0.50	96.2	0.962
1.00	95.0	0.950
3.50	83.1	0.831
6.50	71.4	0.714
19.0	36.4	0.364
30.0	21.7	0.217

T [K]	*k* [$10^{-5}s^{-1}$]	$\Delta G^{\ddagger}$ [kcal/mol]	$_{opt}t_{1/2}$ [h]
298.15	1.421	24.5	13.6
